# Systematic Analysis of Transmitter Coexpression Reveals Organizing Principles of Local Interneuron Heterogeneity

**DOI:** 10.1523/ENEURO.0212-18.2018

**Published:** 2018-10-04

**Authors:** Kristyn M. Lizbinski, Gary Marsat, Andrew M. Dacks

**Affiliations:** Department of Biology, West Virginia University, Morgantown, WV 26505

**Keywords:** Cotransmission, heterogeneity, local interneurons, neuropeptides

## Abstract

Broad neuronal classes are surprisingly heterogeneous across many parameters, and subclasses often exhibit partially overlapping traits including transmitter coexpression. However, the extent to which transmitter coexpression occurs in predictable, consistent patterns is unknown. Here, we demonstrate that pairwise coexpression of GABA and multiple neuropeptide families by olfactory local interneurons (LNs) of the moth *Manduca sexta* is highly heterogeneous, with a single LN capable of expressing neuropeptides from at least four peptide families and few instances in which neuropeptides are consistently coexpressed. Using computational modeling, we demonstrate that observed coexpression patterns cannot be explained by independent probabilities of expression of each neuropeptide. Our analyses point to three organizing principles that, once taken into consideration, allow replication of overall coexpression structure: (1) peptidergic neurons are highly likely to coexpress GABA; (2) expression probability of allatotropin depends on myoinhibitory peptide expression; and (3) the all-or-none coexpression patterns of tachykinin neurons with several other neuropeptides. For other peptide pairs, the presence of one peptide was not predictive of the presence of the other, and coexpression probability could be replicated by independent probabilities. The stochastic nature of these coexpression patterns highlights the heterogeneity of transmitter content among LNs and argues against clear-cut definition of subpopulation types based on the presence of single neuropeptides. Furthermore, the receptors for all neuropeptides and GABA were expressed within each population of principal neuron type in the antennal lobe (AL). Thus, activation of any given LN results in a dynamic cocktail of modulators that have the potential to influence every level of olfactory processing within the AL.

## Significance Statement

Understanding the functional roles of individual local interneurons (LNs) is complex because traits, like transmitter coexpression, are often partially overlapping across the population. Here, we find that single olfactory LNs coexpress neuropeptides from at least four individual peptide families, and that GABA and neuropeptides are partially and heterogeneously coexpressed across the entire population. The stochastic nature of many observed coexpression patterns argues against clear-cut and exclusive definition of subpopulations based on the expression of single neuropeptides. Overall, our results suggest that activation of any given LN causes the release of a variable combination of neuropeptides and GABA that, based on receptor expression, target the input, output, and local processing stages of olfactory coding.

## Introduction

The historical concept of a cell type, propelled by the work of Cajal (1995) and Golgi (1906), suggests that distinct functional classes of neurons can be identified based on their morphology (Ramón et al., 1972; Shepherd, 2015). Yet recent advances in transcriptomics and electrophysiology have revealed that even neurons within a single cell type can still be surprisingly heterogeneous in their synaptic, biophysical, and transcriptional profiles (Cohen et al., 2015; Eddine et al., 2015; Okaty et al., 2015; Li et al., 2017). Local interneurons (LNs) tend to be particularly heterogeneous across many parameters, leading to the identification of numerous LN subtypes within cortex (Flames and Marín, 2005; DeFelipe et al., 2013; Yavorska and Wehr, 2016), hippocampus (Maccaferri and Lacaille, 2003), and spinal cord (Gabitto et al., 2016; Sweeney et al., 2018). For example, two spinal interneuron populations that support different motor output (limb vs thoracic) can be distinguished, and further subdivided, based on transcription factor expression profile (Sweeney et al., 2018). Similarly, 13 distinct groups of GABAergic cortical interneurons exhibit partially overlapping expression of multiple neuropeptides and modulators (Gonchar et al., 2007). Thus, parameters used to classify LN subpopulations can be partially overlapping across functionally distinct subpopulations. Consequently, attempting to assign a unified functional role to subpopulations based on single molecular markers or transmitters is misleading. How then, do we reconcile heterogeneity within cell types?

To determine the organizing principles that govern neuronal heterogeneity, it is critical to use a combinatorial approach, which takes multiple parameters, such as transmitter coexpression, into consideration. The insect antennal lobe (AL), analogous to the olfactory bulb, is an excellent system in which to approach this problem owing to the wealth of information on local interneuron physiology, morphology, and transmitter content combined with its relative numerical simplicity. The olfactory system detects and transforms odor input into meaningful output, ultimately informing an animal’s choice to mate, seek food, or avoid predators (Ache and Young, 2005). Odorants are first detected by olfactory receptor neurons (ORNs), which synapse onto projection neurons (PNs) within substructures called glomeruli that form an odor-topic map within the AL. The input/output relationship between ORNs and PNs is refined by a diverse population of LNs that transform odor information via a variety of mechanisms (Wilson, 2013). In *Manduca sexta*, LNs are primarily inhibitory (Christensen et al., 1993), broadly tuned to odorants, exhibit both inhibitory and excitatory responses, and are highly morphologically and physiologically diverse (Hildebrand et al., 1992; Reisenman et al., 2011). However, there are no correlations between morphology, physiology, and GABA expression in *Manduca* LNs (Reisenman et al., 2011), suggesting a high degree of heterogeneity within this population. Furthermore, in *Manduca*, as well as other insects, AL LNs express a combination of GABA and multiple neuropeptides (Homberg et al., 1990; Schachtner et al., 2004; Utz et al., 2007, 2008; Reisenman et al., 2011; Fusca et al., 2015). Consequently, understanding the functional roles of individual LNs is complex, as we lack a systematic analysis of transmitter coexpression (Nässel, 2018).

We used the olfactory system of *Manduca* to determine if subclasses of LNs have common transmitter profiles. We characterized each pairwise coexpression pattern for GABA and five neuropeptides and found that although almost all peptidergic LNs coexpress GABA, neuropeptide coexpression is heterogeneous across LNs. Using computational modeling, we demonstrate that many coexpression patterns cannot be explained by independent probabilities of expression of each peptide, highlighting that certain pairs of peptides co-occur more (or less) often than by chance. For other pairs, the presence of one peptide was not predictive of the presence of the other, and coexpression probability could be replicated by independent probabilities. The stochastic nature of these coexpression patterns highlights the heterogeneity of transmitter content among LNs and argues against clear-cut and exclusive definition of subpopulation types based on the presence of a single neuropeptide. One possible explanation for this heterogeneity is that principal cell classes within the AL express different GABA and neuropeptide receptors. This would segregate the influence of each modulator across different cell types (Nusbaum et al., 2001, 2017; Tritsch et al., 2016), as is the case for the clock network of *Drosophila melanogaster* (Liang et al., 2017). However, this is not likely to be the case here, as all neuropeptide and GABA_B_ receptors were expressed within every cell class of the AL (ORNs, PNs, and LNs). Overall, our results suggest that activation of any given LN likely releases a variable combination of peptides and GABA to potentially influence every cell class within the AL.

## Materials and Methods

### Animals

*Manduca sexta* were raised at West Virginia University as previously described (Bell and Joachim, 1976; Daly et al., 2013). Equal numbers of unmated adult males and female moths were pooled for all data.

### Immunocytochemistry

Brains were dissected in physiological saline (Christensen and Hildebrand, 1987), fixed in 4% paraformaldehyde overnight at 4°C, and embedded in 5% agarose to be sectioned at 100 µm using a Leica VT 1000S vibratome. Sections were washed in PBS with 1% Triton X-100 (PBST), blocked in PBST and 2% immunoglobulin G (IgG)-free BSA (Jackson Immunoresearch; Cat# 001-000-161), and then incubated in blocking solution with 5 mm sodium azide and primary antibodies. For all rabbit-neuropeptide/mouse-GABA protocols, sectioned tissue was incubated for 48 h at dilutions of 1:3000 and 1:500, respectively. Sections were then briefly washed with PBS and PBST, cleared with ascending glycerol washes, and then mounted on slides with Vectashield (Vector Laboratories; Cat# H-1000). All neuropeptide antibodies used in this study were raised in rabbit. For protocols in which we labeled with two antisera raised in rabbit, we used APEX Antibody Labeling Kits 488, 555, 647 (Invitrogen; Cat# A10468, A10470, A10475, respectively) to directly attach a fluorophore with excitation/emission spectra at different wavelengths to each primary to avoid cross-labeling from a secondary antibody (Bradley et al., 2016). Using the resin tip from the APEX kit, a small amount of the antibody (10–20 µg) was pushed through the resin using an elution syringe, and the reactive dye was prepared using DMSO and a labeling buffer (solutions provided in APEX kit). The reactive dye was eluted through the tip onto the antibody remaining in the resin to covalently bond the fluorescent label to the IgG antibodies. The tip was incubated overnight 4°C or at room temperature for 2 h, and the labeled product was eluted through the tip. Resulting labeled antibody volume of 50 µl in a total volume of 2400 µl was used to label 6 brains at equal dilution of 400 µl per well and incubated for 72 h in 3% Triton X-100 with PBSAT. Sections were then washed and mounted as above.

### Antibody characterization

Specificity controls (including pre-adsorption controls) for the allatostatin-A (AST-A), allatotropin (Mas-AT), tachykinin (TK), and myoinhibitory peptide (MIP) antibodies in *Manduca* brain tissue are described completely in Lizbinski et al. (2016). GABA pre-adsorption controls in *Manduca* AL tissue for the mouse GABA antiserum are described in Bradley et al. (2016). The antibodies used in this study likely cross-react with several isoforms within the same peptide family. Thus, our results can only resolve principles at the level of peptide family and not individual peptide isoforms.

GABA: The GABA antibody (Sigma Aldrich, cat# A2052) was raised in rabbit against GABA coupled to BSA with paraformaldehyde. MIP: Antiserum raised in rabbit against MIP conjugated to thyroglobulin was produced by M. Eckert, Jena, Germany, and provided by C. Wegener, Marburg, Germany (Predel et al., 2001; RRID: AB_2314803). Mas-AT: Antiserum raised in rabbit against *Manduca* allatotropin (Mas-AT) was kindly provided by Dr. J. Veenstra, University of Bordeaux, Talence, France (Veenstra and Hagedorn, 1995; RRID: AB_2313973). AST-A: Antiserum was raised (Reichwald et al., 1994) in rabbit against octadecapeptideallatostatin (Pratt et al., 1991), ASB2 (AYSYVSEYKALPVYNFGL-NH2) of *Diploptera punctata*, and kindly provided by Dr. J. Veenstra. It recognizes AKSYNFGLamide, a form of AST and other AST-like peptides. TK: Antiserum raised in rabbit against locust tachykinin II with bovine thyroglobulin with glutaraldehyde was kindly provided to us by Dr. J. Veenstra (RRID: AB_2341129). FMRF: FMRFamide antiserum was raised against synthetic RF-amide coupled to bovine thyroglobulin with glutaraldehyde and provided by Dr. Eve Marder (Marder et al., 1987). Pre-adsorption controls of the antiserum against synthetic FMRFamide eliminated labeling in larval *Manduca* nervous tissue (Witten and Truman, 1996).

### Confocal microscopy

Image stacks were scanned using an Olympus Fluoview FV1000 confocal microscope with argon and green and red HeNe lasers. Scans were taken at either 800 × 800- or 1024 × 1024-pixel resolution, 1.5 µm between optical sections, using both 20×/0.80 Oil UPlanApo and 40×/1.30 Oil ∞ 0.17/FN 26.5, 80 µm pinhole size, Olympus lenses. Fluoview (FV10-ASW Viewer software, v.4.2b) was also used to set brightness levels, and Corel Draw X4 was used to organize figures.

### Cell counts and coexpression

Images of immunostained brains were exported as .tiff stacks in Fluoview software. Stacks were then imported into VAA3D software (available at https://github.com/Vaa3D/release/releases/; Peng et al., 2010, 2014a,b; Bria et al., 2016) to determine individual cell counts and coexpression cell counts. The number of local interneurons in the lateral cell cluster that express each transmitter were counted in VAA3D (*n* = 6 brains per label combination, 3 brains per sex). We used cell body size and location within the lateral cell cluster to distinguish between LNs and PNs (Homberg et al., 1988). The average and standard deviation of number of cells per AL that expressed a given transmitter were calculated for each combination. Wilcoxon rank sum tests were performed using Prism v.5.01 (GraphPad) to determine if there was any significant difference between the left and the right AL for each brain. Coexpression ratios were determined by dividing the number of cells expressing both an individual neuropeptide and GABA by the total number of cells expressing just the neuropeptide and calculated in Excel. Neuropeptide coexpression ratios were determined in the same manner for every possible pairwise combination using data from peptides stained using the APEX kits. FMRF/MIP coexpression ratios were not calculated, as the APEX kits labeled significantly fewer MIP neurons than all other runs, and therefore the ratios would not have reflected accurate coexpression. Thus, FMRF/MIP coexpression was not used in subsequent models or computational analysis as a constraint or a relationship to replicate. All other neuropeptide/neuropeptide coexpression experiments labeled an accurate number of cell bodies when compared to cell counts from GABA/neuropeptide runs using indirect immunocytochemistry. Cell count totals and standard deviations from APEX kit labeling (Fig. 2) were used in all model iterations, as coexpression ratios were calculated using that data.

### Putative neuropeptide receptor sequence BLAST

We used receptor sequences from closely related invertebrate species to identify putative sequence homologs on *Manduca* scaffolds. Protein sequences from *Drosophila* and other closely related species were identified by annotation (see Table 2) and queried against the *Manduca* genome using tblastn (National Agricultural Library, i5k initiative, https://i5k.nal.usda.gov/Manduca_sexta). Top matches to each receptor sequence in *Manduca* were subsequently queried against the NCBI nr database to confirm their putative annotation as *Manduca* receptor homologs. These sequences were used for primer design for RT-qPCR analysis of putative neuropeptide receptor expression in the antennae, medial and lateral cell clusters, and brain. Sequences that were previously identified in *Manduca* for Mas-ATr and RpS3 (Jiang et al., 1996; Horodyski et al., 2011) were downloaded as FASTA files from NCBI (http://www.ncbi.nlm.nih.gov/gene/?term=) and used to design RT-qPCR primers. Open reading frames were established using ORF Finder at https://www.ncbi.nlm.nih.gov/orffinder/. A recent study partially annotated the *Manduca* genome (Kanost et al., 2016). We used the *Manduca* raw sequence and assembled genome sequence at NCBI Assembly ID GCA_000262585 from Kanost (http://www.ncbi.nlm.nih.gov/assembly/GCA_000262585.1; Kanost et al., 2016) and identified the sequence IDs for each of the transcripts in question (Table 1). None of the putative receptor sequences are currently annotated in NCBI Assembly ID GCA_000262585.

**Table 1.** Neuropeptide cell body totals and percentage coexpression with GABA

Neuropeptide	Average no. of cell bodies in lateral cell cluster	% coexpression with GABA
TK	12.16 ± 0.55	84.6
FMRF	58.16 ± 17.48	92.8
Mas-AT	143.58 ± 24.38	97.2
MIP	150.66 ± 16.79	97.5
AST-A	47.4 ± 12.83	96.7

### Primer design

Open reading frame nucleotide sequences for each receptor, as established above, were used as the basis for primer design for RT-qPCR. Primers were designed using https://www.idtdna.com/calc/Analyzer/Home/Instructions and checked for optimal conditions using OligoAnalyzer 3.1 (https://www.idtdna.com/calc/analyzer). Primers and amplicons were then run through a BLAST of the *Manduca* genome to determine if they matched to the specified sequence and to rule out potential priming mismatches with other parts of the genome. Table 2 lists primer sequences and annealing temperatures. All primers used for RT-qPCR amplified a 90–125-bp stretch of sequence.

**Table 2.** BLAST results for neuropeptide receptor primer design and primer sequences

				Primer sequences		
Receptor	Accession no. of sequence for forward BLAST and origin species	Returned *M. sexta* subject sequence ID and E value	Accession no. of reverse BLAST top hit	Forward	Reverse	Annealing temp. (°C)
TKr	AAA28722.1 (*D. melanogaster*)	Msex2.00568-RA scaffold00007:996602-1079056(+); JH668285.1; E value: e-103	NP_001127749.1 (*Bombyx mori*)	ACAGGTACGTGGCGATAGTG	AGCTGGCACACCAAACAGTA	58.3
FMRFr	AHN57950.1 (*D. melanogaster*)	Msex2.13475-RA scaffold01034:41471-49046(+); JH669301.1; E value: 2e-77	NP_001037007.1 (*Bombyx mori*)	ACCGTGCTCATCCTTACCTC	TGCGGACACACGTGATAGTA	58.3
ASTr	AAG22404.3 (*D. melanogaster*)	Msex2.08175-RB scaffold00218:172215-185483(–); JH668496.1; E value: e-100	ACJ06649.1 (*Spodoptera littoralis*)	ATCTGGCCGTAGCTGATCTT	GCATTACATAAT CCGTTGCG	58.3
MIPr	NP_001108346.1 (*Bombyx mori*)	Msex2.12746-RA scaffold00798:532-18804(+); JH669075.1; E value: e-139	AGE92037.1 (*Spodoptera litura*)	GGGTTCAGGGTACTGTTCGT	GAACAGGAGCACATTCAGGA	58.3
Mas-ATr	ADX66344.1 (*M. sexta*; Horodyski et al., 2011)	JH668656.1	N/A	TTCCTTGGAGACGTGCTGT	ACTTGAACTTGAGCGGG	52
GABA_B_-R1	HG004164.1 (*Heliothis virescens*; at European Nucleotide Archive)	Msex2.03321-RA scaffold00068:510618-566612(–); JH668346.1; E value: 0.0	XM_013339859.1 (*Amyelois transitella*)	TATTTCGGGAATGACTTCTG	TCAATATCATATCCGGCTTC	58.3
RPs3	U12708 (*M. sexta*; Jiang et al., 1996)	JH668297.1	N/A	CATGATCCACTCCGGTGAC	GACCTTAATTCCGAGCACTCC	58.3
vGLUT	FBgn0031424 (*D. melanogaster*; at FlyBase)	JH668481; E value: 5e-18	XM_014627996.1 (Dinoponera quadriceps)	GACCACGACTAATGTGCGGA	CATTGAGTTGACGATCGGCG	58.3

### Real-time quantitative PCR (RT qPCR)

Antennae, medial cell clusters, lateral cell clusters, and brains were collected from 2–6-d-old, unmated, naive adult *Manduca*, and RNA was extracted using a TRIzol reagent (Molecular Research center, Cat# TR 118). Equal numbers of pooled males and females were used for each biological tissue sample for a total of 3 biological samples for each tissue type (*n* = 3 antennae; *n* = 40 medial cell clusters from 20 brains; *n* = 40 medial cell clusters from 20 brains; and *n* = 2 brains). We used the 40s ribosomal protein s3 (RpS3) as our reference gene. RpS3 expression values were consistent across biological replicates. RNA was treated with TURBO DNA-free Kit (Thermo Fisher Scientific, Cat# AM1907) to prevent genomic DNA contamination, and cDNA was synthesized using the SuperScript IV First-Strand Synthesis System (Thermo Fisher Scientific, Cat# 18091050). We performed RT qPCR with the Bio-Rad CFX Connect Real-Time System (Cat #1855201) to determine the relative expression of putative neuropeptide receptors across our tissue samples. Individual samples were prepared by combining prepared cDNA sample, [100 µm] forward and reverse primers, SsoFast EvaGreen Supermix (Bio-Rad, Cat# 1725200), and nuclease-free diH_2_O to a volume of 10 µl. RT^–^ samples, no template controls (NTCs), and positive controls with *Manduca* genomic DNA from the brain were run for every plate. RT^–^ and NTC had no amplification for all receptors and sample types run at 58.3°C (Table 3). Optimal annealing temperatures were determined through a gradient test on genomic DNA to ensure that qPCR on cDNA was performed at optimal temperature. All primer sets, including the reference gene, RpS3, were run using the following protocol [95°C 2 min (95°C 5 s → 58.3°C 30 s) × 39 cycles, 65.0°C 5 s stepped up to 95°C) except for Mas-ATr primers, which were annealed at a temperature of 52°C. All samples for RpS3 were run again at 52°C to ensure accurate calculation of relative expression values for Mas-ATr. Cq values for ANTa (antennae sample a), Mb (Medial cell cluster sample b), and NTC sample for the RpS3 run at 52°C were high (Table 3). However, amplification curves revealed that there were no sharp amplification peaks, and thus high Cq values were due to noise not contamination. High Cq values with nondescript peaks for RpS3 NTCs run at 52°C were considered 0 for ANTa, Mb, and NTC when calculating relative expression.

**Table 3.** Cq Values for all receptors and RpS3 from RT-qPCR

Receptor	RT or RT–	ANTa	ANTb	ANTc	Ba	Bb	Bc	Ma	Mb	Mc	La	Lb	Lc	Genomic
TKr	RT	0	34.1	34.5	31.36	35.35	29.63	38.51	32.18	34.03	39	0	37.59	24.09
	RT–	0	0	0	0	0	0	0	0	0	0	0	0	0 (NTC)
Mas-ATr	RT	35.72	33.65	33.41	32.28	37.32	31.26	39.8	32.42	33.55	35.77	37.6	36.29	30.24
	RT–	0	0	0	0	0	0	0	0	0	0	0	0	39.48 (NTC)
FMRFr	RT	32.43	29.04	29.1	26.56	31.14	25.95	33.01	27.53	28.03	31.57	32.22	30.89	24.85
	RT–	0	0	0	0	0	0	0	0	0	0	0	0	0 (NTC)
MIPr	RT	33.91	31.03	30.69	29.55	33.31	27.94	34.94	28.92	30.15	34.97	35.73	33.12	23.86
	RT–	0	0	0	0	0	0	0	0	0	0	0	0	0 (NTC)
AST-Ar	RT	37.28	32.23	31.23	29.26	23.63	28.17	37.39	31.19	32.39	35.43	37.35	35.14	25.61
	RT–	0	0	0	0	0	0	0	0	0	0	0	0	0 (NTC)
GABA_B_	RT	32.21	29.09	28.62	27.91	31.17	25.97	33.48	27.26	28.51	33.26	35.51	31.97	23.72
	RT–	0	0	0	0	0	0	0	0	0	0	0	0	0 (NTC)
vGlut	RT	x	x	x	24.43	28.40	23.21	x	x	x	32.60	0	31.96	24.82
	RT–	x	x	x	0	0	0	x	x	x	0	0	0	0 (NTC)
RpS3	RT	25.61	22.49	22.16	22.91	26.65	21.41	28.89	23.16	24.04	28.93	28.6	26.04	24.18
	RT–	0	0	0	0	0	0	0	0	0	0	0	0	0 (NTC)
RpS3 (at 52˚C)	RT	24.54	21.85	21.87	23.18	27.04	21.31	28	23.21	24.18	26.68	28.03	25.23	18.29
	RT–	4.41	0	0	39	37.27	0	37.2	5.13	28.94	36.16	38.98	0	5.23 (NTC)

### qPCR relative expression analysis

Raw qPCR data can be found in Table 3. Delta Ct (Ct_receptor_ – Ct_reference gene_) values were calculated for each receptor using RpS3 Ct values as the reference gene and averaged across all biological replicates for brain, lateral cell cluster, medial cell cluster (MCC), and antennae tissue samples. Relative expression levels (2^−ΔCT^) were calculated for all receptors. Ct values ≤37 were considered nondetectable. All graphical representations for receptor qPCR were performed in GraphPad Prism (v. 5.01).

### Computational analysis of transmitter coexpression

We wrote a Matlab program to determine if our observed transmitter coexpression data could be replicated by independent probabilities of expression of each transmitter. Given the known total number of LNs in the lateral cell cluster, and the total number of LNs expressing each neuropeptide from our cell counts, the model determines the probability of a given neuron coexpressing two transmitters. The program is given the average number of cells expressing a given neurotransmitter and then randomly assigns them to one of the cells in the cluster. The model can thus determine the probability of pairwise coexpression (i.e., 100% of TK cells express MIP) in the lateral cell cluster based on chance. Specifically, using our observed data as the backbone of the model, we designed a matrix with 6 columns, 1 per transmitter type (TK, FMRF, Mas-AT, MIP, AST-A, GABA), with a row length of 360 long (the total number of LNs in the lateral cell cluster; Homberg et al., 1988). Within each column, the model randomly distributes the number of cells that express a given transmitter to a row between 1 and 360 (Fig. 3*A*). For example, if we know that 12 cells within the lateral cell cluster are TKergic, the model randomly picks 12 numbers between 1 and 360 in the TK column and marks that cell as TK positive. The number of cells expressing a certain neurotransmitter is chosen probabilistically based on the observed average and standard deviation of the number of neurons that express a given transmitter. With each iteration of the model, the cells that are assigned as transmitter positive within each column are randomized. The model does this with all respective cell count totals for each transmitter column and then calculates the percentage of each transmitter’s expression with another transmitter based on independent expression of each transmitter (across all pairwise comparisons). Standard deviation and percentages of coexpression were recorded across 10,000 iterations of the model. We then compared our observed coexpression percentages to the model’s output to determine if independent probabilities of expression of each transmitter could explain observed coexpression.

The model described above has no initial assumption about the likelihood of coexpression, and only the overall number of cells expressing each of the transmitter is determined initially. We used a similar model to determine if assigning dependent coexpression relationships for specific pairs of transmitters could replicate the coexpression patterns for other transmitter pairs. To do this, we built certain coexpression relationships explicitly as initial assumptions. For example, if we know that on average 100% of TKergic cells are also MIPergic, the program explicitly forces 100% of the cells that are assigned to be TK positive to also be MIP positive. This coexpression relationship is thus no longer determined based on independent expression probabilities like the first version of the script, but rather is an initial assumption: a rule. We can then determine if this rule alone shifts the coexpression of other transmitter types closer to the observed coexpression percentages. We applied these rules one by one (for a total of 94 different models), for every pairwise comparison of coexpression and statistically compared the output of the independent expression model to the output of the rule-based model as well as the observed coexpression patterns we identified with immunocytochemistry. This allowed us to determine if specific coexpression relationships could replicate other coexpression relationships within LNs. The script was run on a Windows 7 desktop, with an Intel Core 17-3770 CPU @ 3.4GHz processor, and a 64-bit operating system.

### Code accessibility

Custom MatLab scripts available at https://www.dacksneuroscience.com/matlab-scripts.html, at https://github.com/lizbinskik2/co-expression-probability, or on request. The code is also available as Extended Data.

10.1523/ENEURO.0212-18.2018.ed1 Extended Data 1 These MatLab scripts calculate the probability of a given neuron coexpressing two neurotransmitters. The script calculates coexpression probabilities for a population of 360 neurons that individually express GABA, MIP, TK, FMRFamide. Code can be altered to include the total number of neurons in a given neural population and the average number (and standard deviation) of neurons that express each transmitter. The probability_script assumes no expression dependencies, and thus the predictions of coexpression are based purely on independent expression probability. Download Extended Data 1, ZIP file.

### Experimental design and statistical analysis

The model outputs a predicted percentage of coexpression for every pairwise coexpression relationship. To statistically determine how well the model replicated observed coexpression percentages, we used standard deviation indices (SDIs) to determine how close the model’s predicted coexpression percentage is to observed probability of coexpression. Similar to a Z-score, this measure is calculated as follows:

$$\text{SDI}=\left({\text{Mean}}_{\text{model}}-{\text{Mean}}_{\text{observed}}\right)/{\text{stdev}}_{\text{greatest}},$$

where Mean_model_ = mean probability of coexpression of any two given neurotransmitters from the model, e.g., mean % TK coexpressed with MIP; Mean_observed_ = mean probability of coexpression of any two given neurotransmitters from the observed coexpression relationships; and stdev_greatest_ = the greatest standard deviation from either the model or observed data for a given coexpression relationship.

Weighted SDIs were calculated to reflect the match between data and model for the overall population of LN by weighting the contribution of each neurotransmitter proportionally to its prevalence:

$$\text{Weighted SDI}=\sum \left[\left({\text{Mean}}_{\text{model}}-{\text{Mean}}_{\text{observed}}/{\text{stdev}}_{\text{greatest}}\right)*\left(n_{\text{coexpressed}}/n_{\text{total}}\right)\right].$$

For example, there are only 12 TK neurons in a total of 360 LNs, but 142 Mas-AT neurons. Therefore, predicting the number of Mas-AT neurons versus TK neurons should carry more weight when determining the accuracy of each model. Weighted SDIs for each coexpression relationship (i.e., weighted SDI for the TK/MIP coexpression) were summed across relationships for an overall measure of the accuracy with which each model iteration replicated observed coexpression patterns.

SDI values can be interpreted by the following scale: 0, perfect consensus between model and experimental data; 1, model results are within one standard deviation of experimental data and thus replicate the data reasonably well; and 2, model results are within two standard deviation of experimental data and thus do not replicate the data accurately. To determine the percent improvement of each model at replicating observed coexpression (Fig. 3*G*), all weighted SDIs were normalized with respect to the weighted SDI of the independent expression model using the following formula:

$$\text{\% improvement from independent expression model}=\left[1-\left({\text{weighted SDI}}_{x}/{\text{weighted SDI}}_{\text{ind}}\right)\right]*100.$$

All statistics were performed in GraphPad prism (v. 5.01).

## Results

The AL of *Manduca* is surrounded by 3 cell clusters that house the cell bodies of projection neurons and LNs. The lateral cell cluster consists of ∼950 cell bodies, including 590 projection neurons and ∼360 total LNs (Homberg et al., 1988), of which ∼170 are GABAergic (Hoskins et al., 1986). *Manduca* LNs are diverse across several traits, with no correlations between physiologic properties, morphologic properties, or GABA expression patterns in LNs (Reisenman et al., 2011). We therefore took a systematic approach to determine if transmitter coexpression could reliably subcategorize and explain the apparent heterogeneity of LN cellular properties.

### Neuropeptide coexpression is highly heterogeneous

**Figure 1.**
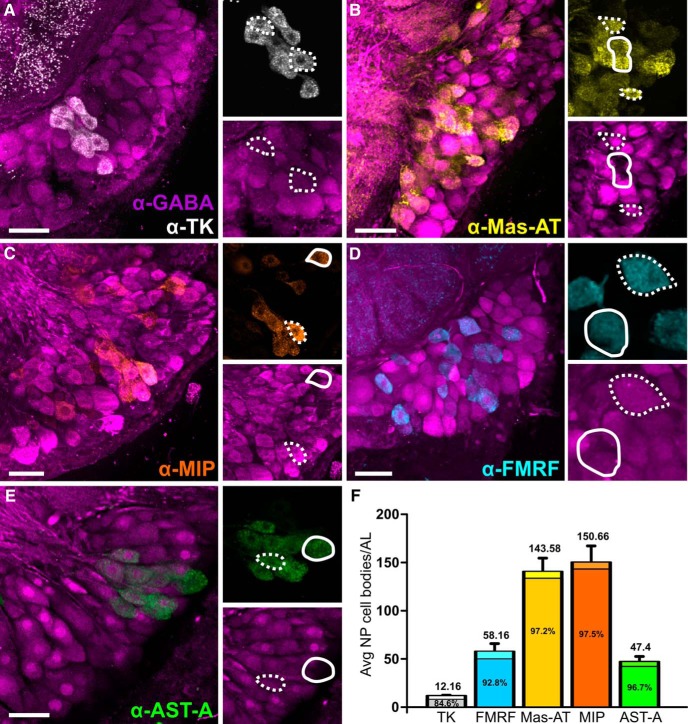
Peptidergic LNs predominantly coexpress GABA. Dashed lines, coexpressed; solid lines, not coexpressed. ***A***, Lateral cell cluster (LCC) labeled for GABA (magenta) and TK (white). ***B***, LCC labeled for GABA (magenta) and Mas-AT (yellow). ***C***, LCC labeled for GABA (magenta) and MIP (orange). ***D***, LCC labeled for GABA (magenta) and FMRFamide (cyan). ***E***, LCC labeled for GABA (magenta) and AST-A (green). ***F***, Bar graph of average number of cell bodies (above bars) that express each transmitter type per AL and the percentage (within bars) of each neuropeptide population per AL that coexpress GABA. See Table 2 for averages and standard deviations. *n* = 6 animals per combination. All scale bars = 50 µm.

**Figure 2.**
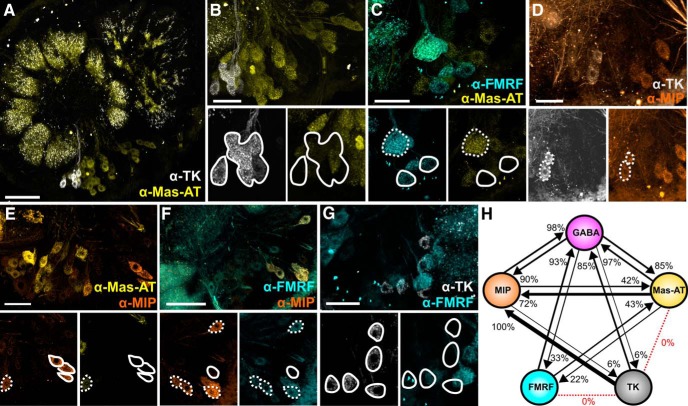
Neuropeptide coexpression is heterogeneous. Dashed lines, coexpressed; solid lines, not coexpressed. Coexpression for ***A***, ***B***: TK (white) and Mas-AT (yellow), ***C***: FMRFamide (cyan) and Mas-AT, ***D***: TK and MIP (orange), ***E***: Mas-AT and MIP, ***F***: FMRFamide and MIP, and ***G***: TK and FMRFamide. All scale bars = 50 µm. ***H***, Schematic representation of transmitter coexpression by LNs. Each circle represents the population of LNs that express a given transmitter. Arrow width and percentage located at arrowhead represent proportion of a given LN type (arrow origin) that also express a second transmitter (arrow destination). FMRFamide and MIP coexpression could not be calculated for technical reasons (see Methods). No TK LNs coexpressed FMRFamide or Mas-AT. Non-GABAergic LNs are not depicted.

We first determined the pairwise coexpression relationships for GABA and multiple neuropeptides TK, FMRF, Mas-AT, MIP, and AST-A in LNs (Fig. 1*A–E*). We chose these neuropeptides because there are available antibodies of sufficient quality, we have performed the proper pre-adsorption controls for each of them, and finally these neuropeptides have the best functional, biochemical, and developmental characterization in *Manduca* as well as other insect species (Carroll et al., 1986; Blackburn et al., 2001; Skaer et al., 2002; Teal, 2002; Utz and Schachtner, 2005; Utz et al., 2007; Yapici et al., 2008; Ignell et al., 2009; Asahina et al., 2014; Ko et al., 2015). All moths were naïve and unmated adults, and equal numbers of males and females were used for each transmitter combination. Using a paired *t* test, we found no significant differences between the left and right lateral cell clusters for all peptides: Mas-AT (*t* = 1.718; df = 5; *p* = 0.1465), MIP (*t* = 0.1056; df = 5; *p* = 0.9200), FMRF (*t* = 0.5324; df = 5; *p* = 0.6172), TK (*t* = 1.085; df = 5; *p* = 0.3276), AST-A (*t* = 0.6407; df = 5; *p* = 0.5499). We also compared counts from male and female moths and, using a paired *t* test, we found no significant differences in cell counts between males and females for MIP (*t* = 1.531; df = 2; *p* = 0.2654), AST-A (*t* = 0.4187; df = 2; *p* = 0.7161), TK (*t* = 0.0000; df = 2; *p* = 1.0), FMRF (*t* = 0.1220; df = 2; *p* = 0.9141). There was a significant difference between males and females in Mas-AT expression (t 11.97; df 2; p 0.0069) with females exhibiting higher Mas-AT expression than males (Male avg: 133, Female avg: 180). Peptidergic LNs predominantly coexpressed GABA (Fig. 1*F*, Table 1), suggesting that LNs can be broadly subdivided into GABAergic/peptidergic and non-GABAergic/non-peptidergic LNs. The non-GABAergic LNs have the potential to be glutamatergic, as RT-qPCR on lateral cell cluster mRNA revealed that the vesicular glutamate transporter (vGLUT) was highly expressed relative to a reference gene (40s ribosomal protein s3; RpS3, see Table 3 for Cq values). A large population of glutamatergic LNs in *Manduca*, in addition to the GABAergic LNs, would be consistent with the organization of the *Drosophila* AL (Das et al., 2011; Liu and Wilson, 2013). We then determined the coexpression ratios (i.e., what percentage of X-expressing neurons coexpress Y) of every pairwise combination of TK, FMRF, Mas-AT, and MIP (Fig. 2*A–G*). There were few consistent coexpression patterns, suggesting that most LNs coexpress multiple neuropeptides to a variable degree (Fig. 2*E*,*G*,*C*). The exception to this rule was TK, which was coexpressed 100% with MIP and never coexpressed with FMRF and Mas-AT (Fig. 2*A*,*B*,*D*,*H*). The 12 TKergic LNs were therefore the only LNs that expressed a consistent transmitter profile. Our results are consistent with other studies of GABA and peptide expression in *Manduca* (Hoskins et al., 1986; Homberg et al., 1990; Utz et al., 2008). Coexpression ratios for each pairwise coexpression relationship (i.e., percentage of neurons that coexpress X and Y) revealed that apart from TK, neuropeptides were coexpressed to a variable degree (Fig. 2*H*).

### Computational analysis of transmitter coexpression reveals that independent expression probability cannot explain observed transmitter coexpression in LNs

**Figure 3.**
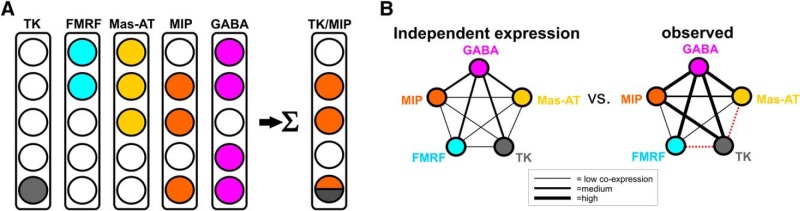
Schematic representations of the computational model used to calculate the probability of LN coexpression patterns. ***A***, Each column represents a transmitter, and the number of rows corresponds to the total number of neurons in the population (reduced in this illustration to 5 total cells for the sake of simplicity, 360 LNs in reality). The number of neurons in each column that are transmitter positive correspond to the average number of neurons (standard deviations built in) that express each transmitter that we observed using immunocytochemistry (see Fig. 1*F* and Table 1 for values). The model then sums across each row in a pairwise fashion to determine the coexpression percentage of a given transmitter pair. For example, for TK/MIP, the model would predict that 1/3 or 33% of MIP neurons (orange) would coexpress TK assuming independent probabilities of expression for each transmitter. ***B***, Schematic representation comparing predicted coexpression percentages from the independent expression model to our observed coexpression patterns. Each circle represents a population of LNs that express a given transmitter. Given the number of neurons that express each individual transmitter (values in Fig. 1*F* and Table 1), the model calculates the probability that a neuron will coexpress two transmitters. Line thickness represents degree to which transmitters are coexpressed. We then compare the predicted coexpression from our independent expression model to our observed coexpression values to see if observed coexpression can be explained based on independent probability of expression.

Two possible scenarios can explain the lack of apparent systematic association between specific neuropeptides coexpressed by LNs. In one scenario, expression of a given neuropeptide is independent of the expression of another, and the likelihood of specific coexpression patterns is equal to the independent probabilities of expression of each transmitter given the number of LNs that express each transmitter. Alternatively, specific pairs of neuropeptides are coexpressed more (or less) often than by chance, and a certain number of such relationships can explain the overall pattern of neuropeptide coexpression. To test these scenarios, we began by using computational modeling to test the hypothesis that coexpression could be explained independent probabilities of expression of each transmitter alone. Given the known total number of LNs in the lateral cell cluster (360; Homberg et al., 1988) and the total number of LNs expressing each neuropeptide (Fig. 1), the model calculates the probability of a neuron coexpressing two transmitters (Fig. 3*A*; see Methods). The model predicts the percentage of neurons that coexpress every pairwise relationship of transmitters in our study. For example, based on the number of LNs that express Mas-AT and the number of LNs that express FMRFamide, and the total number of LNs in the AL, the model determines that 12% of Mas-AT neurons should coexpress FMRF if the probability of expressing the former is independent of the probability of expressing the latter. However, based on our immunocytochemical data, we observed that 22% of Mas-AT neurons coexpress FMRF (Fig 2*H*). We then compared every predicted coexpression ratio from the model (which assumes independent probabilities of expression of each transmitter for each pairwise relationship) to the observed coexpression ratios (Fig. 2*H*) and determined how well the model replicates observed coexpression (Fig. 3*B*). If coexpression probabilities can be replicated by a model that assumes independent expression of each transmitter, then as a result, no organizing coexpression dependencies will be identified.

**Figure 4.**
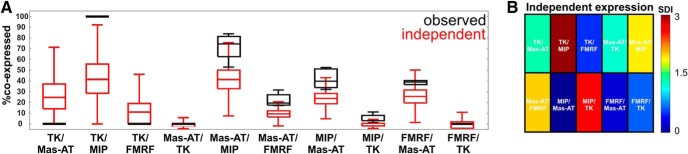
Computational analysis of transmitter coexpression reveals that independent expression probability cannot explain observed transmitter coexpression in LNs. ***A***, Predicted coexpression percentages for every pairwise relationship from the independent coexpression model (red) versus observed coexpression percentages (black). A model that assumes independent probability of coexpression could not replicate observed coexpression percentages. ***B***, Statistical comparison of the independent coexpression model’s prediction versus observed coexpression reveals that independent probability of coexpression alone cannot replicate observed LN coexpression patterns. Each colored rectangle represents an individual pairwise relationship (e.g., TK/Mas-AT). SDIs were calculated for every pairwise relationship to determine how closely the model could replicate observed coexpression. An SDI of 0 (blue) denotes no statistical difference between observed coexpression and predicted coexpression from the model, thus representing coexpression relationships that the model was able to replicate very well. SDI values >1 indicate a poor match between the model and observed values.

We found that most coexpression relationships were not replicated by a model assuming independent transmitter expression (Fig. 4*A*; independent expression model). To statistically measure how well our model replicated observed coexpression patterns, we then used an SDI for every predicted pairwise coexpression relationship versus observed coexpression. An SDI score of 0 indicates that our simulation perfectly recapitulates observed coexpression patterns, whereas an SDI score >1 indicates poor performance of the model. Each predicted coexpression ratio from the model was compared to the observed coexpression ratios, and a SDI was calculated [SDI = (Mean_model_ – Mean_observed_)/stdev_greatest_]. SDI scores for every pairwise coexpression relationship were statistically weighted (see Methods), such that coexpression relationships that included a larger proportion of the total LN population carried more weight. SDI scores revealed that while an independent-expression model could replicate some coexpression relationships (with a weighted SDI of 1.49), independent expression alone does not accurately replicate the observed coexpression (Fig. 4*B*).

### A few specific coexpression constraints allow replication of overall coexpression patterns

**Figure 5.**
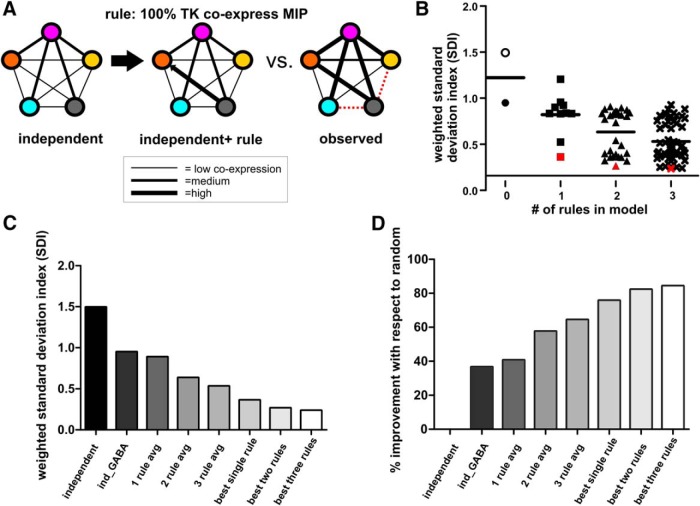
A few specific coexpression constraints allow replication of overall coexpression patterns. ***A***, Model constraints are applied to explicitly set the probability of a coexpression relationship to its observed value. In this example, a constraint is set in which 100% of TK LNs coexpress MIP, while leaving the remaining relationships to emerge through probabilistically independent expression. This model is then compared to observed coexpression data. ***B***, Specific rules outperform others at replicating observed coexpression patterns. Open circle represents model run with total number of LNs set to 360. Closed symbols represent models runs with total number of neurons set to the total number of GABAergic LNs (∼170). Red denotes standout iterations of the model that best replicated observed coexpression. The single rule that shifted the prediction closest to observed coexpression was when the proportional relationship between MIP/Mas-AT was set as a static rule in the model (red square). The two rules that shifted the prediction closest to observed coexpression were MIP/Mas-AT and TK/Mas-AT. The three rules that shifted the prediction closest to observed coexpression were TK/Mas-AT + Mas-AT/MIP + FMRF/Mas-AT. ***C***, Weighted SDI values for various model iterations. The model improves as more rules are added. ***D***, Percentage improvement of each model’s predictive power with respect to the independent expression model. Both GABA constraint and MIP/Mas-AT rule drastically improved the model’s ability to replicate coexpression patterns. Note that the MIP/Mas-AT rule model even outperformed the average prediction of all models containing three rules.

Since the independent expression of each transmitter did not replicate the overall probabilities of coexpression patterns, we next sought to identify which coexpression relationship must be adjusted to replicate the overall structure of coexpression. We implemented, in our model, rules according to which the probability of expression of a transmitter is dependent on the expression of another transmitter (Fig 5*A*), thereby explicitly setting the probability of coexpression to its observed value (Fig. 2*H*). Therefore, the model contains a set number of coexpression relationships in the form of rules (for example, 42% of MIPergic LNs coexpress Mas-AT as observed from our immunocytochemistry), while leaving the rest of the relationships to emerge through probabilistically independent expression. We tested 94 different model iterations, each containing different combinations of coexpression rules to determine which combinations of rules best replicated overall observed coexpression (Fig. 5*B*). This allowed us to identify predictive coexpression relationships in an unbiased manner. As expected, the ability of the model to replicate observed coexpression patterns improved as more rules were added, as shown by weighted SDIs from all model iterations (Fig. 5*B–D*).

**Figure 6.**
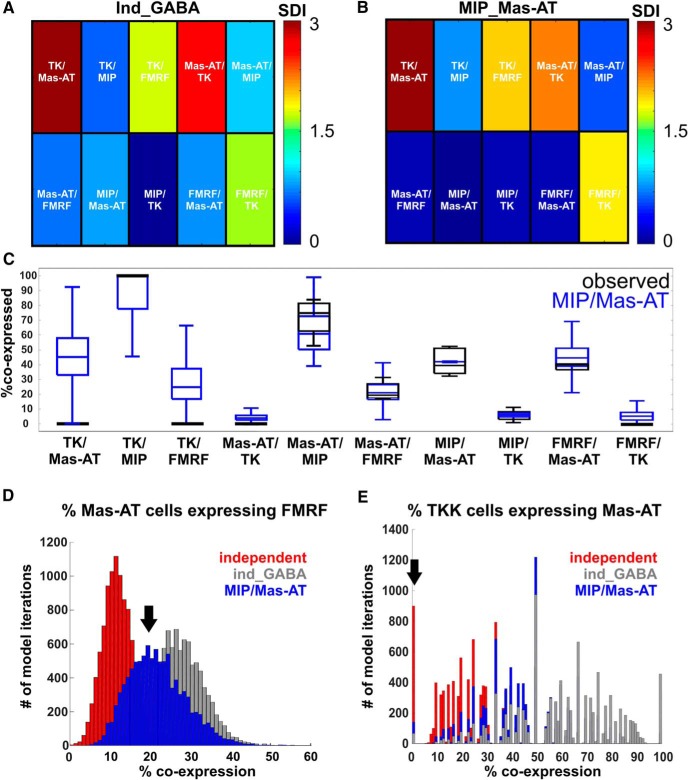
MIP/Mas-AT coexpression rule best biases the model to replicate observed coexpression patterns. ***A***, Reducing the total number of neurons in the model to the total number of GABAergic LNs (170) improves model performance. SDI = 0 (blue) denotes no statistical difference between observed coexpression and predicted coexpression. SDI > 1 indicates a poor match between the model and observed values. ***B***, Constraining the model based on MIP/Mas-AT coexpression causes the model to reliably replicate many observed coexpression patterns. A model following this single rule outperformed the average of all models containing three set coexpression rules. ***C***, All predicted pairwise coexpression percentages from the model following the MIP/Mas-AT rule (blue) versus observed coexpression percentages (black). ***D***, Neither independent (red), nor ind_GABA (gray) models reliably replicated observed coexpression patterns (Mas-AT/FMRF used as an example). However, the MIP/Mas-AT (blue) constraint best replicates observed coexpression patterns (denoted by black arrow). ***E***, Observed TK coexpression patterns (TK/Mas-AT used an example) were not reliably replicated by any model iteration; independent expression model prediction (red), ind_GABA model (gray), and MIP/Mas-AT model prediction (blue).

However, some combinations of rules outperformed others. We first constrained the total number of cells in the model to the total number of GABAergic LNs (∼170 cells instead of 360 total LNs), as the presence of GABA is a reliable predictor of peptide expression observed in this study. This constraint outperformed the independent-expression model, had a weighted SDI of 0.94, and accurately replicated more coexpression patterns (Fig. 6*A*). This suggests that much of the diversity of neuropeptide coexpression can be constrained to the subpopulation of GABAergic LNs in our study. All remaining model iterations were constrained to the total number of GABAergic LNs [Fig. 5*B*; filled-in symbols indicate models where total number of LNs = 170 (with stdev) GABAergic neurons]. Unexpectedly, one particular model that contained only 1 coexpression rule outperformed most models that were constrained by 2 and 3 rules (red square, Fig. 5*B*). When the proportion of MIPergic LNs expressing Mas-AT is set to its observed value (42%), the model replicated the highest number of coexpression relationships of all models with 1 rule (Fig. 6*B–D*), yielded the lowest weighted SDI (0.36), and outperformed the average of models with 1 rule (lower 95% CI of mean: 0.6551, upper 95% CI of mean: 0.9862), 2 rules (lower 95% CI of mean: 0.5450, upper 95% CI of mean: 0.7240), and even 3 rules (lower 95% CI of mean: 0.4736, upper 95% CI of mean: 0.5897). This was surprising, because it suggested that replicating observed coexpression patterns did not require all coexpression relationships to be fixed, revealing specific proportional relationships that may be may be predictive of overall observed coexpression patterns.

The only set of coexpression patterns that could not be replicated reasonably well in the model that included the GABA and the MIP/Mas-AT rules (as described above) involves TK. The 12 TK LNs (Lizbinski et al., 2016) follow a strict all-or-none neuropeptide coexpression pattern (100% coexpression with MIP and 0% coexpression with Mas-AT or FMRF). Consistent with our data, TK LNs in the AL of the moth *Heliothis virescens* also do not coexpress FMRF or Mas-AT (Berg et al., 2007). This coexpression pattern cannot be replicated through independent expression models, even when several other rules are considered (Fig. 6*E*). These coexpression patterns are so clear-cut that they set TK apart from other transmitters observed in this study.

### GABA_B_ and neuropeptide receptors are expressed across all principal neuron types of the AL

**Figure 7.**
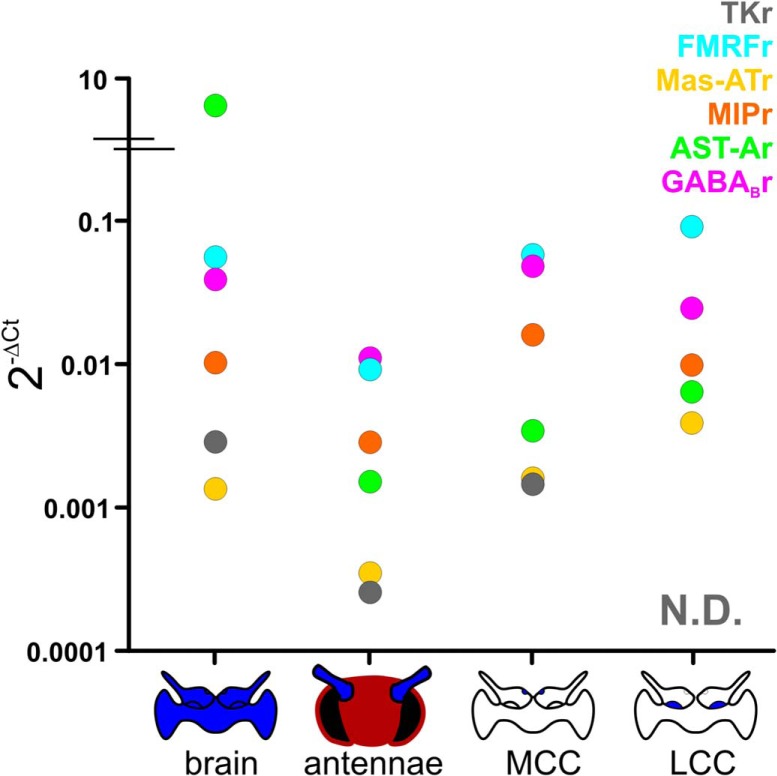
Neuropeptide and GABA_B_ receptor expression across principal neuron types of the AL. Relative receptor expression for Mas-ATr, MIPr, ASTr, FMRFr, GABA_B_r are present in all tissue types and therefore expressed in ORNs, LNs, and PNs in varying expression levels. Cartoons on the *x*-axis represent the tissue type (blue) used to extract mRNA from each population of principal olfactory cell types. TK was not detectable (N.D.) in lateral cell cluster mRNA and therefore not detectable in LNs. RpS3 was used as the reference gene. See Table 2 for primer sequences and Table 3 for raw Cq values for all receptors.

It may be unnecessary to tightly regulate coexpression of neuropeptides in specific subpopulations of LNs simply because specific classes of AL neurons express different sets of neuropeptide receptors. Thus, the heterogeneous transmitter profiles of individual LNs may not matter functionally, because the impact of individual peptides within a modulatory cocktail of many peptides may be segregated due to neuron class-specific expression of each receptor. For instance, if ORNs express the MIP receptor and PNs express the Mas-AT receptor, the influence of these two neuropeptides could differentially target input and output of the network, rather target the same neuron, resulting in different consequences on the network. However, this does not appear to be the case in this network, as we did not find differential expression of the receptors for the peptides examined in this study between ORNs, LNs, and PNs. We first identified transcripts from the *Manduca* genome (Kanost et al., 2016) with high sequence identity to neuropeptide receptors identified from reference genomes in closely related species (Table 2). Then, using RT-qPCR, we determined the relative expression of five neuropeptide receptors (Mas-AT, MIP, AST-A, FMRF, TK) and the GABA_B_ receptor in mRNA from the antennae (which house ORNs), the medial cell cluster (which houses only PNs), the lateral cell cluster (which houses LNs and PNs), and whole brains (as a positive control). Although the receptors for Mas-AT, MIP, AST-A, FMRF, and GABA_B_ were detected in all four tissue types, the TK receptor was not detected in the lateral cell cluster (Fig. 7; for raw qPCR data, see Table 3). This suggests that TKergic LNs differ from other LNs in both their coexpression patterns and their postsynaptic targets. Although we could not assess receptor expression on a neuron-by-neuron basis, our results suggest that a single LN releasing neuropeptides from at least four individual peptide families can have a powerful effect on the network, potentially affecting all three major cell classes in the AL.

## Discussion

**Figure 8.**
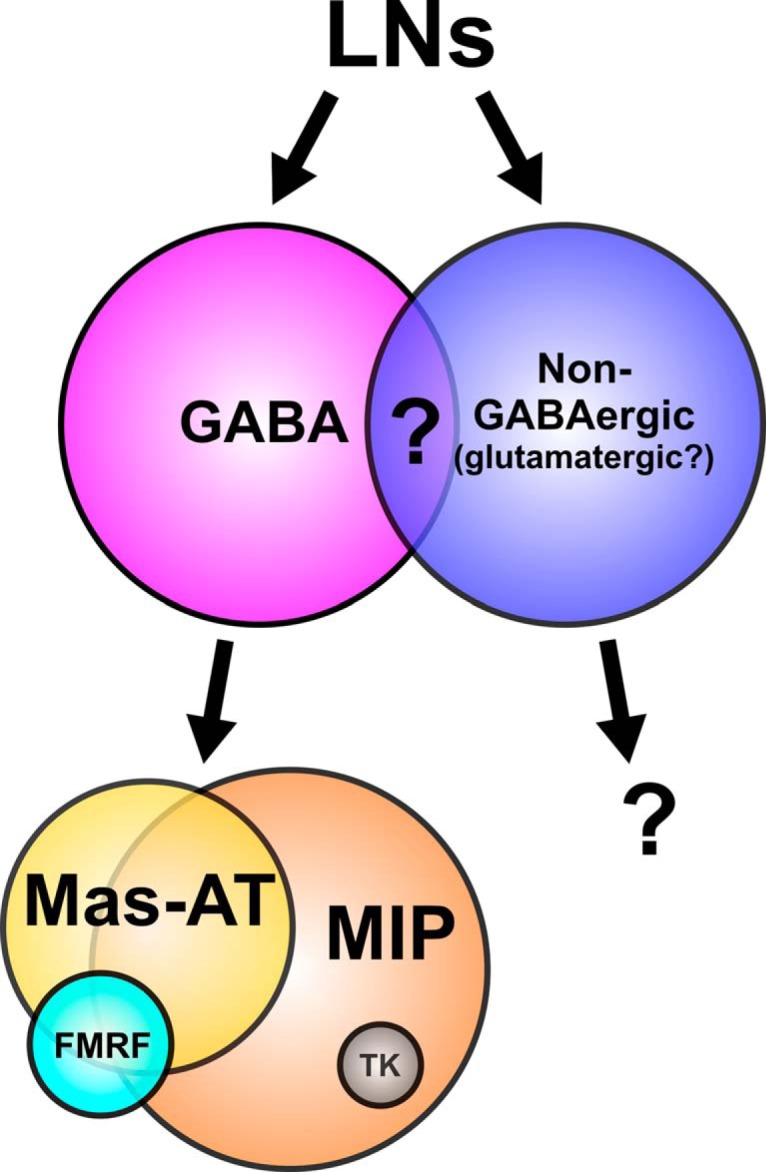
Heterogeneous transmitter coexpression in LNs blurs subdivisions. While LNs can be broadly subdivided based on small transmitter [GABA versus non-GABAergic (glutamatergic?)], coexpression within the GABAergic class reveals that LNs subclasses cannot be identified on individual transmitter expression alone. Neuropeptide coexpression in the AL is both heterogeneous and partially overlapping across the entire population rather than consistent within specific subpopulations of LNs.

Broad neuronal classes are surprisingly heterogeneous across many parameters, and subclasses often exhibit partially overlapping traits including transmitter coexpression. Our goal was to determine organizing principles of LN heterogeneity. Our results suggest that neuropeptide coexpression in the AL is both heterogeneous and partially overlapping across the entire population rather than consistent within specific subpopulations of LNs (Fig. 8). Thus, peptidergic modulation cannot be considered within the context of single neuropeptides, as activation of any given LN results in a dynamic cocktail of modulators that have the potential to influence every level of olfactory processing within the AL. Specifically, we find that transmitter profile is heterogeneous across LNs, with individual olfactory LNs capable of expressing the main inhibitory transmitter GABA and peptides from at least four families, and few instances in which transmitters are consistently coexpressed. Observed coexpression patterns cannot be explained by independent probabilities of expression of each transmitter (Fig. 4). Our analyses point to three organizing principles that, once taken into consideration, allow replication of overall coexpression structure: (1) peptidergic neurons are highly likely to coexpress GABA; (2) the probability of expressing Mas-AT is dependent on MIP expression; and (3) the all-or-none coexpression patterns of TKergic neurons with several other neuropeptides (MIP, FMRF, and Mas-AT). For other pairs, the presence of one transmitter was not predictive of the presence of the other, and thus coexpression probability could be replicated by independent probabilities. The stochastic nature of these coexpression patterns argues against clear-cut, exclusive definition of subpopulations based on the presence of single neuropeptides. Furthermore, the receptors for GABA and all neuropeptides in this study were expressed within each population of principal neuron type in the AL (Fig. 7), suggesting that peptides released from LNs potentially influence every level of olfactory processing within the AL. Overall, we demonstrate that peptide expression is partially overlapping across LNs, and thus subpopulations of LNs cannot be functionally defined based on the presence of single peptides. Furthermore, the influence of peptides are not segregated based on cell class-specific receptor expression. Thus, co-release of peptides and GABA likely mediates a complex mix modulation to control the dynamic range of the AL, providing multiple mechanisms to alter olfactory processing.

Heterogeneous transmitter coexpression is a common theme within GABAergic LNs across vertebrates and invertebrates alike (Homberg et al., 1990; Maccaferri and Lacaille, 2003; Flames and Marín, 2005; Utz et al., 2008; Carlsson et al., 2010; DeFelipe et al., 2013; Siju et al., 2014; Binzer et al., 2014; Gabitto et al., 2016; Yavorska and Wehr, 2016; Diesner et al., 2018a). MALDI-TOF spectrometry revealed that at least 12 known peptides are expressed in developing *Manduca* ALs (Utz et al., 2007), suggesting that coexpression patterns are likely even more complex than detailed here. Furthermore, the antibodies used in this study recognize multiple isoforms of peptides within the same family (i.e., FMRF has multiple isoforms), and thus there are almost certainly more organizational principals underlying heterogeneous peptide expression than discussed here. Other insects including mosquitos (Siju et al., 2014), other species of moths (Berg et al., 2007; Diesner et al., 2018a), beetles (Binzer et al., 2014), and fruit flies (Carlsson et al., 2010; Hussain et al., 2016; Croset et al., 2018) express a large number of peptides within their olfactory systems, suggesting that peptides likely play an important yet functionally underexplored role in shaping olfactory responses. One exception to the theme of heterogeneous coexpression was that the TK neurons differed in their patterns of coexpression from other peptidergic LNs. All TK LNs coexpressed MIP, and none coexpressed FMRF or Mas-AT, suggesting that TK LNs are primarily inhibitory, as TK and MIP receptors are inhibitory in *Drosophila* (Yapici et al., 2008; Ignell et al., 2009; Ko et al., 2015). Furthermore, TK receptor transcripts were not detected in lateral cell cluster mRNA and thus not in LNs, although TK/MIPergic LNs could still influence LNs via GABA_B_ and MIP receptor. In *Drosophila melanogaster*, TK mediates presynaptic gain control on ORNs (Ignell et al., 2009), and TKr expression in *Manduca* ORNs is consistent with this finding. This suggests that TK LNs may play a role distinct from other LNs in olfactory processing, which could include presynaptic gain control.

Very few non-GABAergic LNs coexpress the neuropeptides we examined here; however, they are still a sizeable proportion of the total number of LNs and likely as heterogeneous as GABAergic LNs. We did not definitively identify the transmitter released by these LNs; however, we did detect the expression of vGlut mRNA within the lateral cell cluster (Fig. 7), making glutamate a candidate transmitter for the non-GABAergic LNs. Similar to GABAergic LNs, glutamatergic LNs in *Drosophila* are particularly diverse in their morphology (Das et al., 2011) but appear to differ from GABAergic LNs in their synaptic targets by predominantly affecting PNs (Liu and Wilson, 2013), while GABAergic LNs in *Drosophila* affect ORNs, LNs, and PNs (Wilson and Laurent, 2005; Olsen and Wilson, 2008; Root et al., 2008; Hong and Wilson, 2015). Future studies should confirm whether the non-GABAergic population observed here are truly glutamatergic.

The probability of expression of certain transmitters appears to be dependent on one another. In particular, we showed that the probability of expressing Mas-AT is dependent on the expression of MIP (Fig. 6). While the goal of our study is not to determine the developmental mechanisms that underlie coexpression, it is important to note that developmental mechanisms of peptidergic regulation most certainly shape observed heterogeneous coexpression. For instance, the molting hormone 20-hydroxyecdysone induces Mas-AT expression in LNs and other neuropeptides in the AL of *Manduca* (Utz and Schachtner, 2005; Utz et al., 2007), implying that coexpression patterns may reflect extrinsic developmental cues that guide the development of specific peptide-expressing LNs. Furthermore, both Mas-AT– and MIP-expressing LNs arise slightly before and during the formation of glomeruli, suggesting that temporal expression patterns of these peptides may play a role in the development of AL structure and function (Utz et al., 2007). Interestingly, the model constraint best able to replicate observed coexpression across all LNs in our study was the proportional relationship between MIP/Mas-AT-expressing neurons.

However, the developmental mechanisms that control peptide expression in LNs of *Manduca* are unknown. In *Drosophila,* the transcription factor DIMMED targets many genes involved in peptide expression (Hewes et al., 2003, 2006; Gauthier and Hewes, 2006; Park et al., 2008b; Park and Taghert, 2009; Hadzic et al., 2015) and dense core vesicle production (Hamanaka et al., 2010; Park et al., 2014). DIMMED likely acts in a combinatorial manner with other cell-specific transcription factors to determine peptide expression in individual neurons (Liu et al., 2016; Stratmann and Thor, 2017). Although DIMMED does not target any single neuropeptide gene (Hadzic et al., 2015), other transcription factors do regulate subtype-specific neuropeptide expression (Allan et al., 2003; Berndt et al., 2015). While DIMMED-positive neurons coexpress multiple peptides, not all peptidergic neurons express DIMMED (Park et al., 2008a; Diesner et al., 2018b), and the role of DIMMED in *Manduca* has not been established. Regardless, a similar combinatorial transcriptional code could underlie the heterogeneity of peptide expression observed here. Furthermore, in cortex, LN subtypes arise from unique progenitors (Anderson et al., 1997; Wichterle et al., 2001; Nery et al., 2002; Kepecs and Fishell, 2014), and their diversity is shaped by additional factors (Flames and Marín, 2005) including neural activity (Patz et al., 2004; De Marco García et al., 2011), transcription factor expression (Mayer et al., 2018; Sweeney et al., 2018), and growth factors (Huang et al., 1999). Similarly, GABAergic and glutamatergic LNs in *Drosophila* arise from distinct neuroblasts (Das et al., 2008, 2011), and glomerular innervation patterns of LNs require ORN axons during development (Chou et al., 2010), suggesting that heterogeneity of LNs may be due in part to distinct origins and/or activity of other neurons in the network.

Our study reveals some expression codependencies, but also highlights the apparent stochastic nature of other coexpression patterns. There are several examples of biological systems in which features such as gene expression in *E. coli* clones (Elowitz et al., 2002; Raj and van Oudenaarden, 2008; Huh and Paulsson, 2011), behavior (Honegger and de Bivort, 2018), or anatomic layout (Caron et al., 2013) appear to be randomly structured or stochastic. For example, random combinations of AL PNs from different glomeruli converge and synapse on individual mushroom body Kenyon cells in *Drosophila* regardless of anatomy, developmental origin, or odor tuning, thus abandoning the odor-topic organization of the AL (Caron et al., 2013). Because of the stochastic heterogeneity of some transmitter coexpression patterns, our results suggest that the presence of single peptides should not be used to functionally define classes of neurons. Additionally, this stochasticity suggests that LNs may not functionally require fixed complements of transmitters.

We found that a single neuropeptide has the potential to simultaneously target every principal neuron type, as all neuropeptide receptors were expressed by populations of ORNs, LNs, and PNs. This network-wide convergence of peptidergic modulation demonstrates that individual LNs do not differentially target principal neuron type based on differences in postsynaptic receptor expression. This further supports the idea that LN activation may serve to regulate multiple processing stages within the olfactory network by simultaneously targeting AL input, output, and local processing. However, individual neurons within each principal AL neuron type may exhibit differential receptor expression, as we were not able to assess receptor expression at the level of individual neurons. Future studies should determine if neuropeptide receptor expression is as heterogeneous as neuropeptide coexpression itself, as there are likely subpopulations of neurons that exhibit differential receptor expression. This may be further complicated, as neuropeptide receptor expression can be regulated by physiological state, as observed for the role of hunger (Ko et al., 2015; Min et al., 2016) or mating state in *Drosophila* (Hussain et al., 2016). Peptide expression itself may be similarly regulated, as observed in feeding state of *Aedes aegypti* (Christ et al., 2017) or mating state of *Agrotis ipsilon* moths (Diesner et al., 2018a). All moths in our study were naïve and unmated; however, this does not rule out the potential for physiologic state to affect peptide expression in the AL.

Activation of even a single LN can mediate a complex mix of inhibition and/or excitation that varies in time course and strength due to the co-release of the small-transmitter GABA and a heterogeneous mix of peptides. LNs, apart from TK LNs, coexpressed multiple peptide families and GABA that activate both inhibitory (TK, sNPF, and sex-peptide/MIP; Yapici et al., 2008; Ignell et al., 2009; Asahina et al., 2014; Ko et al., 2015) and excitatory (Mas-AT and FMRF; Horodyski et al., 2011; Lenz et al., 2015; Ormerod et al., 2015) receptors via a mix of ionotropic and metabotropic signaling. Furthermore, AL neurons express both the GABA_a_ and GABA_B_ receptors, and the effects of GABA_B_ receptor activation are far shorter-lasting relative to neuropeptide receptors (Salio et al., 2006). Thus, small-transmitter and peptide coexpression expands the temporal scale with which a single neuron can alter network processing. However, it is unclear whether LNs employ bulk and/or restricted synaptic release of peptides, making the spatial scale of their influence unknown. Finally, the network may need to be more strongly activated (i.e., by higher concentrations of odors or increased length of odor-stimuli) for LNs to release neuropeptides owing to the different calcium binding affinities of distinct synaptotagmins associated with small clear vesicles and dense-core vesicles (Saraswati et al., 2007; Li et al., 2009). Thus, the consequences of LN activation and peptidergic modulation may depend more on the degree of network activity than the identity of any singular LN that is activated. Overall, this heterogeneous cocktail of peptides likely provides the AL with flexible options to up- or down-regulate olfactory processing over a variety of time frames and spatial scales within the context of ongoing network activity.

Within the AL, combined GABAergic and peptide release from LNs could potentially play a variety of functional roles including autoinhibition, lateral excitation or inhibition, disinhibition, and even odor-specific processing. For example, lateral input from LNs scales with overall network activity as a means to control the dynamic range of the network and avoid response saturation of PNs (Olsen and Wilson, 2008; Root et al., 2008). Additionally, some glomeruli are more subject to inhibition than others simply because of differences in glomerulus-specific, non-uniform LN innervation (Wilson and Laurent, 2005; Chou et al., 2010) and ORN GABAb receptor expression (Root et al., 2008). As a result, the processing of specific odors differs in the degree of insulation from ongoing activity in the olfactory system, and specific glomeruli are therefore more (or less) insulated from presynaptic gain control mediated by both GABA (Root et al., 2008) and, potentially, neuropeptides (Ignell et al., 2009; Ko et al., 2015; Hussain et al., 2016). Spatial activation of *Drosophila* LNs is also odor-specific and heterogeneous, with LNs responding to either single or multiple odors (Ng et al., 2002). The nonuniform innervation and heterogeneous odor-evoked responses of LNs suggests that the activation of LNs is a complex combinatorial process resulting in glomerular-specific local processing. In *Manduca*, most GABAergic LNs are wide-field and heavily ramify all glomeruli, suggesting that the consequences of GABAergic LN activation cannot be fully segregated based on odor identity. However, a small subset of GABAergic and non-GABAergic LNs exhibit restricted glomerular arborizations, only innervating a small subsection of the AL (Reisenman et al., 2011). Consequently, activation of morphologically restricted LNs may disinhibit or inhibit other LNs from neighboring glomeruli in an odor-specific manner to increase or decrease odor salience by altering the output of PNs (Hildebrand et al., 1992; Christensen et al., 1993). While no correlations between morphology (wide-field vs restricted), physiology, odor-response profile, and transmitter content have been identified in *Manduca* LNs (Reisenman et al., 2011), it could be that wide-field versus restricted LNs exhibit distinct and predictable combinations of peptides. These potential network consequences are likely applicable across insect species, as LN heterogeneity is a recurring theme. Using physiology paired with hierarchical clustering based on morphology, multiple *Drosophila* LN subtypes exhibit broad correlations between morphology, physiology, and genetic classes (Chou et al., 2010). However, LNs within the “patchy” cell type exhibit highly variable innervation patterns, and considerable diversity exists even within other LN subtypes (Chou et al., 2010). Additionally, morphologically and functionally distinct classes of LNs exist in honeybees (Schäfer and Bicker, 1986; Flanagan and Mercer, 1989; Fonta et al., 1993; Sun et al., 1993; Bornhauser and Meyer, 1997; Seidel and Bicker, 1997; Galizia and Kimmerle, 2004; Dacks et al., 2010) and cockroaches (Malun, 1991; Distler and Boeckh, 1997; Loesel and Homberg, 1999; Husch et al., 2009a, b; Fusca et al., 2013, 2015; Neupert et al., 2018). Ultimately, determining the roles of individual peptides will be challenging, as complex patterns of coexpression must be integrated with knowledge of functionally distinct subtypes of LNs.

Reconciling within-cell-type heterogeneity represents an ongoing challenge. Similar to LNs across taxa and brain region, *Manduca* LNs are highly heterogeneous across many parameters. This heterogeneity provides multiple coding strategies and mechanisms to neurons within the same population, expanding the role single neurons play in altering network function. The link between heterogeneous response properties and neural coding has been studied in a wide range of systems (Chelaru and Dragoi, 2008; Marsat and Maler, 2010; Ogawa et al., 2011; Pitkow and Meister, 2012; Ahn et al., 2014); however, the systematic analysis of heterogeneous traits such as transmitter coexpression has not been as extensively explored. Here, we show that traits such as transmitter coexpression are partially overlapping across the entire LN population. Ultimately, our results demonstrate that peptidergic modulation cannot be considered within the context of single neuropeptides, as activation of any given LN results in a dynamic cocktail of modulators that have the potential to influence every level of olfactory processing within the AL.

10.1523/ENEURO.0212-18.2018.ed2 Extended Data 2 Similarly, the script_Predict_FMRF_Mas-AT script allows you to explicitly set the coexpression probability of two given transmitters to its observed value. For example, you may know that 30% of FMRF-expressing neurons also express the transmitter Mas-AT based on physical data from immunocytochemistry. This relationship is then set as an explicit rule and coexpression dependency, leaving the remaining coexpression relationships to emerge based on independent probability of expression. This allows you to determine if there are dependent coexpression relationships in your population of neurons that may be predictive of other relationships in the population in an unbiased manner. Download Extended Data 2, ZIP file.

## Synthesis

Reviewing Editor: Darcy Kelley, Columbia University Decisions are customarily a result of the Reviewing Editor and the peer reviewers coming together and discussing their recommendations until a consensus is reached. When revisions are invited, a fact-based synthesis statement explaining their decision and outlining what is needed to prepare a revision will be listed below. The following reviewer(s) agreed to reveal their identity: Joachim Schachtner. Note: If this manuscript was transferred from JNeurosci and a decision was made to accept the manuscript without peer review, a brief statement to this effect will instead be what is listed below.

This paper seeks to characterize organizing principles behind inhibitory interneuron types in the antennal lobe of adult moths (Manduca sexta) by examining the co-expression of immunoreactivity against 5 different neurally expressed peptides in GABA positive neurons. The authors used a computational approach to analyze co-expression patterns and employed qPCR to determine whether receptors for GABA and these neuropeptides were expressed in antennal lobe cell clusters containing only olfactory receptor neuron (ORNs), interneurons (LN), or a mixture of LNs and principal neurons. The reviewers agree that the data are sound and carefully analyzed. However, the questions addressed are not framed broadly and the functional implications of the findings are not discussed clearly enough (though the additional detail in lines 607 - 633 is a step in the right direction; see comments below). In addition, the specificity of the antisera needs to be spelled out more clearly given recent information on peptide isoforms. All of these concerns could be addressed in a re-written version of the paper. Some suggestions for revision preceding re-review are detailed below.

General comments

One framework for understanding neural functions is “cell type”: the idea that the NS consists of developmentally and genetically specified classes of neurons that can be distinguished by their morphology (cell size, dendritic and axonal arbors), connectivity, molecular identity- including the expression of neurotransmitters and NT receptors - and electrophysiological characteristics. These features can then be used to predict the functions of the neural circuits within which individual neurons operate, decoding sensory information, for example, or specifying different forms of motor output (e.g. gait or other muscle activity patterns). This prevailing idea must originally spring from Cajal's observations of canonical cell morphologies across vertebrates (e.g. the Purkinje cell) and the subsequent identification of the roles played by these distinct cell types in neural circuits. Where this idea breaks down, however, is in the circuitry sub-served by inhibitory interneurons, especially those (as here) that participate in sensory decoding but perhaps also in other groups, such as the inhibitory interneurons of the spinal cord, whose actual function in the circuit continues to elude even the most sophisticated computational analyses.

The insect antennal lobe provides a useful system in which to approach this problem. It functions in decoding odors and that function is subserved by connectivity between olfactory receptor neurons, (ORNs) interneurons (LNs: primarily inhibitory) and the projections of the principal neurons (PN) that LNs innervate to other targets such as the mushroom bodies. Across insects, the LNs express a large number of peptides some of which might serve as neuromodulators or neurotransmitters (though not well-established for all) and it is logical to ask (as in this paper) whether peptide co-expression can provide organizing principles for the way that the circuitry supports the task of distinguishing between odors. These issues have been tackled in Drosophila which has the advantage of genetic approaches including the visualization of morphologies and connectivity, the identification of stem cell origins using lineage markers and the ability to delete specific, genetically-specified classes. While it is also possible to record from antennal lobe neurons in these flies, the neurons are much smaller. Of especial relevance to this manuscript is the Chou et al. paper in Drosophila which addressed the organization of antennal lone LNs using a different approach. The advantage of moths is cell size, facilitating electrophysiological characterizations, and resulting greater wealth of functional information as to functional cell types. Information is also available for cockroaches, for example, and including this information on aspects in which Manduca detail is lacking would broaden interest the paper's audience.

In addition, the current manuscript version does not provide enough background for the reader to discern the framework within which the study was carried out. For example, the system itself, the various glomeruli, the neuron types, their connectivity and what is known re odor specificity and odor responses is not sufficiently described. In this era of mice and fruit flies, it is unfortunately the case that the general readership of eNeuro cannot be expected to appreciate the advantages of this system without some background. The more general background - is there really a “cell type” whose molecular identity defines a circuit function - would also help the reader to appreciate the authors' approach.

Specific comments on revised text

Author's additional text in the revision.

Abstract: and Discussion “Overall, heterogeneous co-expression and network-wide convergence of peptidergic modulation suggests that LNs act as a diverse collective to broadly regulate the tone of the olfactory system.”

Given that LNs do not uniformly innervate all glomeruli in the AL, the conclusion that they act as a “diverse collective” (suggesting different cell types act in unison” seems unlikely. Also what does “tone of the olfactory system” mean?

Introduction “The consequence of this heterogeneity is that different coding strategies, transmitter profiles, and synaptic connectivity of LNs provide a variety of mechanisms that expand the computational capacity of a network.” [This sentence is repeated verbatim at the end of the Introduction]

This sentence implies that some mechanisms have been identified in other systems as well as computational capacity (not clear what this is). Can any - for example the recent Sweeney et al. paper on spinal cord - be applied to the results of this paper?

Results “We chose these neuropeptides because there are available antibodies of sufficient quality, we have performed the proper pre-adsorption controls for each of them, and finally these neuropeptides have the best functional, biochemical, and developmental characterization (Utz and Schachtner, 2005; Utz et al., 2007; Yapici et al., 2008; Ignell et al., 2009; Asahina et al., 2014; Ko et al., 2015).”

Peptide profiling in Manduca reveals that, for example, the antibody used by the authors recognizes all four isoforms (cleaved products of a larger precursor) of FMRF. As these isoforms constitute additional possibilities for diversity, this general limitation with regards to the peptides and antisera should be highlighted in the Methods section. It is not possible to determine from antibody labelling which isoform is co-expressed with other peptides only which peptide class. The number of neuropeptides recognized by the antisera used is at least eight (see line 47 in the Abstract).

Only if all the neuropeptides examined here have been shown to function as neurotransmitters or neuromodulators in Manduca, is the term “transmitter” (e.g. lines 49 and 50 and elsewhere in the Abstract) warranted. For the authors' stated purpose (examining the organizational principles) it doesn't actually matter whether MIP et al. are transmitters; they are being used primarily to see if diversity codes can be predicted combinatorially. The authors could, for example, have used transcription factors or calcium binding proteins instead. However, if the peptide does have modulatory or transmitter functions which affect the reception of the GABergic signal (as suggested by the qtPCR results) then the combinatorial code might describe some important aspect of olfactory processing (for example shortening the IPSP for a particularly salient odor).

Discussion

“Furthermore, because ORNs, PNs and LNs all express every receptor examined here, LNs are not separating the network consequences of each transmitter based on cell class-specific receptor expression. Thus, co-release of peptides and GABA likely mediate a complex mix modulation to control the dynamic range of the AL, providing multiple mechanisms to flexibly alter olfactory processing.”

As noted above, the projection patterns of LNs are not uniform. In flies, for example, some avoid specific glomeruli while others (“patchy”) appear to tile the post-synaptic space. It is also not clear, exactly, what “network consequences of each transmitter” means, nor “dynamic range of the AL.” How exactly might diversity confer flexibility?

“Additionally, this stochasticity suggests that LNs may not functionally require fixed compliments of transmitters and thus should be considered a heterogeneous collective rather than attempting to assign functional roles based on individual peptide expression.”

The universe of peptides expressed in the LN is not yet known; many of the substances profiled in Utz et al. 2007 (100) using mass spec are unknown and the authors did not use antisera directed against all of the 12 identified peptides, raising the possibility that some additional organizing principles (other than peptide + GABA and MIP plus allostatin) might exist.

One argument in the Discussion is that most of the LNs are wide field and therefore activation of LNs leads very likely to broad modulation of the network. However, some groups of glomeruli are innervated by subgroups of LNs whose activation would affect only these glomeruli. Broadening the discussion beyond Manduca to include the many insect systems (Drosophila, Aedes, other moths beetles) with large numbers of different peptides in ALs would strengthen the Discussion. Additional issues are the possibility of subnetworks at the sender/receiver level (not clear from the PCR data) and of centrifugal neurons.

Specific comments on the text:

Introduction

Lines 98 to 99

Citation missing: Schachtner et al. 2004. Development and steroid regulation of RFamide immunoreactivity in antennal lobe neurons of the sphinx moth Manduca sexta. J exp Biol. 207: 2389-2400.

Lines 101 to 102

The citations refer to other species than M. sexta but the two papers by Utz et al. are on M. sexta. Appropriate citations: Heliothis: Berg et al. 2007, 2009; Drosophila: Carlsson et al. 2010. Cockroach: Fusca et al. 2015. Siju et al. 2014 does not refer to colocalization.

Line 105

“Nässel” or “Naessel” rather than “Nassel”

Lines 124 to 125 and 593 to 594

The sentence suggests that every AL neuron expresses those receptors.

Methods section

All the antisera cross react to peptide c-terminally ends characterstiic for each peptide family. Within the same species these peptide isoforms stem from the same gene: AST-As, AT, TKRPs and MIP but not RFamides. Typically antisera label more than one peptide.

Lines 133-134

Were the moths adult? N for each sex?Y “Cell counts and co-expression” are 3 females and 3 males.Is there a difference in terms of cell numbers?

Line 157 From a reviewer:

Perhaps abbreviate as AST-A, as there are other ASTs.

Line 158

Other authors refer to AT when they abbreviate Allatotropin, why use ATR? For TKK, the authors use TK or if they address the peptide family: TKRP. For the FMRFa one might use -RFa which might be more appropriate, as there is probably only -FLRFa in M. sexta.

Lines 181 to 183

Additional information on objectives resolution of the scans, pinhole size, and distance between optical sections would be helpful.

Line 168

as would be information on VAA3D software

Line 188 :6 brains per label combination were used; .each brain contains 2 ALs. CDDoes the M mean: one LC per brain? 2 LCs per brain? If the analysis included both left and right LC,are there laterality differences?

Results

Lines 352 to 353

It is unlikely that there are 360 LNs of which exactly 164 are GABA positive.

Lines 361 to 365 See previous comments re antisera

Lines 365 to 366

Adult moths?

“integrated at the level of the network, rather than within the same neuron”? What does this mean explicitly?

Lines 491 to 493

Belongs in the Discussion.

Figure 1 F and table 1

The numbers for Mas-AT differ from Utz et al. 2008. Why this difference?

Discussion

Lines 497 to 499

Differentiating between the principal transmitter GABA and the neuropeptides would make it much easier to follow arguments throughout the Discussion centering on the peptides.

Lines 511 to 513

Why not have functionally defined LNs with overlapping peptide expression? For example,suppose that of 90 -RFamide LNs, 20 contain in addition AT, 20 AST-A and the rest AST-A plus say two more peptides. Are not these group functionally defined?

Line 514

What are “network consequences”?

From line 521

Citations that strenghten this argument:

e.g. Drosophila: Carlsson et al. 2010, Aedes aegypti: Siju et al. 2014, Tribolium: Binzer et al. 2014, Agrotis: Diesner et al. 2018)

Line 589 to 591

Meaning of this sentence is not clear.

Line 618

“driven harder” is unclear.

Line 621 and 623

Is .....overall network “activity” and not “activation” meant here?

Lines 627 to 634

This well differentiated conclusion is not yet reflected, overall, in the paper.

Lines 646 to 647

Correct but in only one aspect

## Author Response

Response to Reviews for manuscript eN-TNWR-0212-18X “Systematic analysis of transmitter co-expression reveals organizing principles of local interneuron heterogeneity”

Dear Colleagues,

Thank you for your constructive comments on our manuscript. We have responded to every comment in-line. The comments from reviewers are bolded and in font style Calibri. Our responses are denoted by a yellow highlighted carat (&gt;), and are in Times new Roman. We have also included the line number of specific changes within the main article text.

---------------------------------------------

Synthesis of Reviews:

Computational Neuroscience Model Code Accessibility Comments for Author (Required):

No changes needed

Significance Statement Comments for Author (Required):

Acceptable

Comments on the Visual Abstract for Author (Required):

NA

Synthesis Statement for Author (Required):

This paper seeks to characterize organizing principles behind inhibitory interneuron types in the antennal lobe of adult moths (Manduca sexta) by examining the co-expression of immunoreactivity against 5 different neurally expressed peptides in GABA positive neurons. The authors used a computational approach to analyze co-expression patterns and employed qPCR to determine whether receptors for GABA and these neuropeptides were expressed in antennal lobe cell clusters containing only olfactory receptor neuron (ORNs), interneurons (LN), or a mixture of LNs and principal neurons. The reviewers agree that the data are sound and carefully analyzed. However, the questions addressed are not framed broadly and the functional implications of the findings are not discussed clearly enough (though the additional detail in lines 607 - 633 is a step in the right direction; see comments below). In addition, the specificity of the antisera needs to be spelled out more clearly given recent information on peptide isoforms. All of these concerns could be addressed in a re-written version of the paper. Some suggestions for revision preceding re-review are detailed below.

General comments

One framework for understanding neural functions is “cell type”: the idea that the NS consists of developmentally and genetically specified classes of neurons that can be distinguished by their morphology (cell size, dendritic and axonal arbors), connectivity, molecular identity- including the expression of neurotransmitters and NT receptors - and electrophysiological characteristics. These features can then be used to predict the functions of the neural circuits within which individual neurons operate, decoding sensory information, for example, or specifying different forms of motor output (e.g. gait or other muscle activity patterns). This prevailing idea must originally spring from Cajal's observations of canonical cell morphologies across vertebrates (e.g. the Purkinje cell) and the subsequent identification of the roles played by these distinct cell types in neural circuits. Where this idea breaks down, however, is in the circuitry sub-served by inhibitory interneurons, especially those (as here) that participate in sensory decoding but perhaps also in other groups, such as the inhibitory interneurons of the spinal cord, whose actual function in the circuit continues to elude even the most sophisticated computational analyses.

The insect antennal lobe provides a useful system in which to approach this problem. It functions in decoding odors and that function is subserved by connectivity between olfactory receptor neurons, (ORNs) interneurons (LNs: primarily inhibitory) and the projections of the principal neurons (PN) that LNs innervate to other targets such as the mushroom bodies. Across insects, the LNs express a large number of peptides some of which might serve as neuromodulators or neurotransmitters (though not well-established for all) and it is logical to ask (as in this paper) whether peptide co-expression can provide organizing principles for the way that the circuitry supports the task of distinguishing between odors. These issues have been tackled in Drosophila which has the advantage of genetic approaches including the visualization of morphologies and connectivity, the identification of stem cell origins using lineage markers and the ability to delete specific, genetically-specified classes. While it is also possible to record from antennal lobe neurons in these flies, the neurons are much smaller. Of especial relevance to this manuscript is the Chou et al. paper in Drosophila which addressed the organization of antennal lone LNs using a different approach. The advantage of moths is cell size, facilitating electrophysiological characterizations, and resulting greater wealth of functional information as to functional cell types. Information is also available for cockroaches, for example, and including this information on aspects in which Manduca detail is lacking would broaden interest the paper's audience.

In addition, the current manuscript version does not provide enough background for the reader to discern the framework within which the study was carried out. For example, the system itself, the various glomeruli, the neuron types, their connectivity and what is known re odor specificity and odor responses is not sufficiently described. In this era of mice and fruit flies, it is unfortunately the case that the general readership of eNeuro cannot be expected to appreciate the advantages of this system without some background. The more general background - is there really a “cell type” whose molecular identity defines a circuit function - would also help the reader to appreciate the authors' approach.

&gt; A section has been added to the introduction to prime the reader on the organization, connectivity and advantages of the M. sexta olfactory system (Lines 98-115) as well as general introduction to the idea of “cell type” (Lines 79-95). We also added a section to the discussion which frames our results in the broader context of insects whose nervous systems express many peptides (Lines 550-554) and in which functional subtypes of LNs have been identified (Lines 686-697). Finally, we have significantly expanded the functional implications of our findings in the discussion (Lines 640-685).

Specific comments on revised text

Abstract: and Discussion

“Overall, heterogeneous co-expression and network-wide convergence of peptidergic modulation suggests that LNs act as a diverse collective to broadly regulate the tone of the olfactory system.”

Given that LNs do not uniformly innervate all glomeruli in the AL, the conclusion that they act as a “diverse collective” (suggesting different cell types act in unison” seems unlikely. Also what does “tone of the olfactory system” mean?

&gt; The language has been changed to exclude “diverse collective” as well as “tone of the olfactory system”. We have reformed our argument in the discussion to exclude these terms.

Introduction

“The consequence of this heterogeneity is that different coding strategies, transmitter profiles, and synaptic connectivity of LNs provide a variety of mechanisms that expand the computational capacity of a network.” [This sentence is repeated verbatim at the end of the Introduction]

This sentence implies that some mechanisms have been identified in other systems as well as computational capacity (not clear what this is). Can any - for example the recent Sweeney et al. paper on spinal cord - be applied to the results of this paper?

&gt; We have changed this paragraph and framed it within the results of the Sweeney et al 2018 paper and the concept of cell type Lines 79-95 “The historical concept of a cell-type, propelled by the work of Cajal and Golgi, suggests that distinct functional classes of neurons can be identified based on their morphology. Yet recent advances in transcriptomics and electrophysiology have revealed that even neurons within a single cell-type can still be surprisingly heterogeneous in their synaptic, biophysical and transcriptional profiles (Cohen et al., 2015; Eddine et al., 2015; Okaty et al., 2015; Li et al., 2017). Local interneurons (LNs) tend to be particularly heterogeneous across many parameters, leading to the identification of numerous LN subtypes within cortex (Flames and Marin, 2005; DeFelipe et al., 2013; Yavorska and Wehr, 2016), hippocampus (Maccaferri and Lacaille, 2003) and spinal cord (Gabitto et al., 2016; Sweeney et al., 2018). For example, two spinal interneuron populations which support different motor output (limb vs. thoracic) can be distinguished, and further subdivided, based on transcription factor expression profile (Sweeney et al., 2018). Similarly, 13 distinct groups of GABAergic cortical interneurons exhibit partially overlapping expression of multiple neuropeptides and modulators (Gonchar et al., 2007). Thus, parameters used to classify LN sub-populations can be partially overlapping across functionally distinct sub-populations. Consequently, attempting to assign a unified functional role to sub-populations based on single molecular markers or transmitters is misleading. How then, do we reconcile heterogeneity within cell-types?.”

Results

“We chose these neuropeptides because there are available antibodies of sufficient quality, we have performed the proper pre-adsorption controls for each of them, and finally these neuropeptides have the best functional, biochemical, and developmental characterization (Utz and Schachtner, 2005; Utz et al., 2007; Yapici et al., 2008; Ignell et al., 2009; Asahina et al., 2014; Ko et al., 2015).”

Peptide profiling in Manduca reveals that, for example, the antibody used by the authors recognizes all four isoforms (cleaved products of a larger precursor) of FMRF. As these isoforms constitute additional possibilities for diversity, this general limitation with regards to the peptides and antisera should be highlighted in the Methods section. It is not possible to determine from antibody labelling which isoform is co-expressed with other peptides only which peptide class. The number of neuropeptides recognized by the antisera used is at least eight (see line 47 in the Abstract).

&gt; Yes, this limitation is now listed in the methods (164-166) and discussion (Lines 547-550). In the previous version we stated “MALDI-TOF spectrometry revealed that at least 12 known peptides are expressed in developing Manduca ALs (Utz et al., 2007), suggesting that co-expression patterns are likely even more complex than detailed here.” But we have also added a statement to this new revision highlight that there are likely even more organizing principles not uncovered here due to the complexity of the peptidergic system Lines 547-550. The full details of preadsorption controls for each peptide antibody are listed in our manuscript “The anatomical basis for modulatory convergence in the antennal lobe of Manduca sexta” Lizbinski et al 2016, J. Comp. Neurol.

Only if all the neuropeptides examined here have been shown to function as neurotransmitters or neuromodulators in Manduca, is the term “transmitter” (e.g. lines 49 and 50 and elsewhere in the Abstract) warranted. For the authors' stated purpose (examining the organizational principles) it doesn't actually matter whether MIP et al. are transmitters; they are being used primarily to see if diversity codes can be predicted combinatorially. The authors could, for example, have used transcription factors or calcium binding proteins instead. However, if the peptide does have modulatory or transmitter functions which affect the reception of the GABergic signal (as suggested by the qtPCR results) then the combinatorial code might describe some important aspect of olfactory processing (for example shortening the IPSP for a particularly salient odor).

&gt; Each neuropeptide examined here has been shown to be functionally active within Manduca (Lines 371-377). While some of these effects are neuroendocrine in nature, the presence of neuropeptide receptor homologues within principal olfactory cell types highly suggests that these peptides are active within the nervous system. We believe that changing every instance of the word ‘transmitter’ to ‘peptides’ will be confusing to the reader and thus we maintain the use of transmitter when discussing broad concepts of co-expression. In specific places where we are discussing only peptides, we have changed ‘transmitter’ to ‘neuropeptide’ to be clearer. We discuss the functional implications of GABA and neuropeptide release in our discussion (Lines 640-659).

Discussion

“Furthermore, because ORNs, PNs and LNs all express every receptor examined here, LNs are not separating the network consequences of each transmitter based on cell class-specific receptor expression. Thus, co-release of peptides and GABA likely mediate a complex mix modulation to control the dynamic range of the AL, providing multiple mechanisms to flexibly alter olfactory processing.”

&gt; We changed this section to read “Overall, we demonstrate that peptide expression is partially overlapping across LNs and thus sub-populations of LNs cannot be functionally defined based on the presence of single peptides. Furthermore, neuropeptide and GABAB receptors were expressed within each population of principal neuron type in the AL because ORNs, the influence of peptides are not segregated based on cell class-specific receptor expression. Thus, co-release of peptides and GABA likely mediate a complex mix modulation to control the dynamic range of the AL, providing multiple mechanisms to alter olfactory processing”

As noted above, the projection patterns of LNs are not uniform. In flies, for example, some avoid specific glomeruli while others (‘patchy’) appear to tile the post-synaptic space. It is also not clear, exactly, what “network consequences of each transmitter” means, nor “dynamic range of the AL.” How exactly might diversity confer flexibility?

&gt; We had added a section in our discussion about the functional consequences of diversity, non-uniform innervation patterns of LNs, as well as the variability of ‘patchy” LNs (Lines 660-697). We have removed the use of “network consequences of each transmitter”. Finally, the term dynamic range refers to the range of activation states that a network can exhibit. Because networks can shift between different states (from complete quiescence through to a variety of different network states) this term was coined to encompass the plastic manner in which the overall activity in a network changes. It is a common term used in neuroscience to refer to the possible range of activity that could be observed from an entity, be it a single neuron, a circuit or a complex network.

“Additionally, this stochasticity suggests that LNs may not functionally require fixed compliments of transmitters and thus should be considered a heterogeneous collective rather than attempting to assign functional roles based on individual peptide expression.”

The universe of peptides expressed in the LN is not yet known; many of the substances profiled in Utz et al. 2007 (100) using mass spec are unknown and the authors did not use antisera directed against all of the 12 identified peptides, raising the possibility that some additional organizing principles (other than peptide + GABA and MIP plus allostatin) might exist.

&gt; Yes, definitely. In the previous version of this manuscript we state “MALDI-TOF spectrometry revealed that at least 12 known peptides are expressed in developing Manduca ALs (Utz et al., 2007), suggesting that co-expression patterns are likely even more complex than detailed here.” In this revised version we added another statement “Furthermore, the antibodies used in this study recognize multiple isoforms of peptides within the same family (i.e. FMRF has multiple isoforms), and thus there are almost certainly more organizational principals underlying heterogeneous peptide expression than discussed here.” (Lines 547-550)

One argument in the Discussion is that most of the LNs are wide field and therefore activation of LNs leads very likely to broad modulation of the network. However, some groups of glomeruli are innervated by subgroups of LNs whose activation would affect only these glomeruli.

&gt; We have significantly altered and expanded our discussion of this point and odor-specific modulation Lines (660-697).

Broadening the discussion beyond Manduca to include the many insect systems (Drosophila, Aedes, other moths beetles) with large numbers of different peptides in ALs would strengthen the Discussion.

&gt; The discussion has been expanded to frame our results within the broader context of insect systems (Lines 550-554).

Additional issues are the possibility of subnetworks at the sender/receiver level (not clear from the PCR data) and of centrifugal neurons.

&gt; Yes, we agree. There are likely subnetworks that exhibit different combinations of receptors as we note Lines 506-509 “Although we could not assess receptor expression on a neuron-by-neuron basis, our results suggest that a single LN releasing at least four individual transmitters can have a powerful effect on the network, potentially affecting all three major cell classes in the AL.”

And lines 629-639 “ However, individual neurons within each principal AL neuron type may exhibit differential receptor expression, as we were not able to assess receptor expression at the level of individual neurons. Future studies should determine if neuropeptide receptor expression is as heterogeneous as neuropeptide co-expression itself as there are likely sub-populations of neurons that exhibit differential receptor expression. This may be further complicated as neuropeptide receptor expression can be regulated by physiological state, as observed for the role of hunger (Ko et al., 2015; Min et al., 2016) or mating state in Drosophila (Hussain et al., 2016). Peptide expression itself may be similarly regulated, as observed in feeding state of Aedes aegypti (Christ et al., 2017) or mating state of Agrotis ipsilon moths (Diesner et al., 2018b). All moths in our study were naïve and unmated, however this does not rule out the potential for physiological state to affect peptide expression in the AL.”

As for centrifugal neurons, we did not assess their role in this paper. However, we do discuss the role of centrifugal neurons targeting intrinsic local interneurons extensively in our recent review Lizbinski and Dacks 2018 “Intrinsic and Extrinsic Neuromodulation of Olfactory Processing”.

Specific comments on the text:

Introduction

Lines 98 to 99

Citation missing: Schachtner et al. 2004. Development and steroid regulation of RFamide immunoreactivity in antennal lobe neurons of the sphinx moth Manduca sexta. J exp Biol. 207: 2389-2400.

&gt; This citation has been added

Lines 101 to 102

The citations refer to other species than M. sexta but the two papers by Utz et al. are on M. sexta. Appropriate citations: Heliothis: Berg et al. 2007, 2009; Drosophila: Carlsson et al. 2010. Cockroach: Fusca et al. 2015. Siju et al. 2014 does not refer to colocalization.

&gt; The Utz, and Siju citations were removed and the Berg, Carlsson and Fusca citations have been added

Line 105

“Nässel” or ‘Naessel’ rather than ‘Nassel’

&gt; The citations have been changed to include the appropriate spelling of Nässel

Lines 124 to 125 and 593 to 594

The sentence suggests that every AL neuron expresses those receptors.

&gt; This sentence has been changed to: “However, this is not likely to be the case here, as all neuropeptides and GABA receptors were expressed within every cell class of the AL (ORNs, PNs and LN).”

And

“We found that a single neuropeptide has the potential to simultaneously target every principal neuron type as all neuropeptide receptors were expressed by populations of ORNs, LNs and PNs.”

With the caveat: “However, individual neurons within each principal AL neuron type may exhibit differential receptor expression, as we were not able to assess receptor expression at the level of individual neurons. Future studies should determine if neuropeptide receptor expression is as heterogeneous as neuropeptide co-expression itself as there are likely sub-populations of neurons that exhibit differential receptor expression.”

Methods section

All the antisera cross react to peptide c-terminally ends characterstiic for each peptide family. Within the same species these peptide isoforms stem from the same gene: AST-As, AT, TKRPs and MIP but not RFamides. Typically antisera label more than one peptide.

&gt; We have added the statement: “The antibodies used in this study likely cross-react with several isoforms within the same peptide family. Thus, our results can only resolve principles at the level of peptide family and not individual peptide isoforms” Lines 164-166, and in the discussion “Furthermore, the antibodies used in this study recognize multiple isoforms of peptides within the same family (i.e. FMRF has multiple isoforms), and thus there are almost certainly more organizational principals underlying heterogeneous peptide expression than discussed here.” Lines 548-550

Lines 133-134

Were the moths adult? N for each sex?Y “Cell counts and co-expression” are 3 females and 3 males.Is there a difference in terms of cell numbers?

&gt; All moths were adults, N = 3 was used for each sex, for a total of 6 brains per transmitter combination. Using a paired t-test, we found no significant differences in cell counts between males and females for MIP (t=1.531; df=2;p= 0.2654) , AST-A (t=0.4187; df=2; p= 0.7161), TK (t=0.0000; df=2; p=1.0), FMRF (t=0.1220; df=2; p=0.9141). There was a significant difference between males and females in Mas-AT expression (t=11.97; df=2; p = 0.0069). For our computational model we used the pooled average of males and females as well as the standard deviation. The model randomized the total # of cells it set per loop (10,000 times), so we believe pooling males and females even for Mas-AT covers the full spectrum of possible cell counts found in both male and female ALs. Lines 378-385

Line 157 From a reviewer:

Perhaps abbreviate as AST-A, as there are other ASTs.

&gt; All instances of AST were replaced with AST-A

Line 158

Other authors refer to AT when they abbreviate Allatotropin, why use ATR? For TKK, the authors use TK or if they address the peptide family: TKRP. For the FMRFa one might use -RFa which might be more appropriate, as there is probably only -FLRFa in M. sexta.

&gt; All abbreviations for peptides are now changed in the text and figures to be consistent with other authors. However, the antibody we used to stain for FMRF (provided by Eve Marder) is referred to as anti-FMRFamide in “Developmental plasticity of neuropeptide expression in motoneurons of the moth, Manduca sexta: steroid hormone regulation” Witten and Truman, 1996 in which staining was completely abolished by synthetic FMRFamide. So to remain consistent with their nomenclature, we maintain the use of FMRF throughout our manuscript.

ATR -&gt; Mas-AT, TKK -&gt; TK, AST -&gt; AST-A

Lines 181 to 183

Additional information on objectives resolution of the scans, pinhole size, and distance between optical sections would be helpful.

&gt; This information was added Lines 188-191 “Scans were taken at either 800x800 or 1024x1024 pixel resolution, 0.5 to 1.5 um between optical sections, using both 20x/0,80 Oil UPlanApo, and 40x/1.30 Oil ∞ 0.17/FN 26.5, 80um pinhole size, Olympus lenses.

Line 168

as would be information on VAA3D software

&gt; Citations and information regarding VAA3d software was added Line 196

Line 188 :6 brains per label combination were used; .each brain contains 2 ALs. CDDoes the M mean: one LC per brain? 2 LCs per brain? If the analysis included both left and right LC, are there laterality differences?

&gt; There is one lateral cell cluster per AL. Our analysis was based on the average # of LNs per lateral cell cluster in one AL. The averages and standard deviations for each peptide total were calculated based on the cell counts from the left and right lateral cell cluster in each AL of each animal. The left and right totals were added for each animal, and divided by two to find the average number of cell bodies per lateral cell cluster. The total number of cells per LC were then averaged across all six animals and the standard deviation calculated. Furthermore, the average number (and standard deviation) of cell bodies per peptide/per lateral cell cluster was included in the model to account for variation between animals and ALs (avg and stdev found in Figure 1). Using a paired t-test we found no significant differences between the left and right lateral cell clusters for all peptides: Mas-AT (t=1.718; df=5; p=0.1465), MIP (t=0.1056; df=5; p=0.9200), FMRF (t=0.5324;df=5; p=0.6172), TK (t=1.085;df=5; p=0.3276), AST-A (t=0.6407;df=5; p=0.5499) (Lines 378-381)

Results

Lines 352 to 353

It is unlikely that there are 360 LNs of which exactly 164 are GABA positive.

&gt; We changed this sentence to read “The lateral cell cluster consists of ~950 cell bodies, including 590 projection neurons and all ~360 total LNs (Homberg et al., 1988), of which ~170 are GABAergic (Hoskins et al., 1986).” (Lines 362-363)

Lines 361 to 365 See previous comments re antisera

&gt; The wording throughout the paper and here been changed to reflect the comments about antisera. See above comments

Lines 365 to 366

Adult moths?

&gt; Yes, the sentence is changed to “All moths were naïve and unmated adults, and equal number of males and females were used for each transmitter combination” (Lines 377-378)

“integrated at the level of the network, rather than within the same neuron”? What does this mean explicitly?

&gt; We changed this sentence to “For instance, if olfactory receptor neurons (ORNs) express the MIP receptor and projection neurons (PNs) express the ATR receptor, the influence of these two neuropeptides could differentially target input and output neurons, rather than simultaneously target the same neuron, resulting in drastically different consequences on the network.” (Lines 491-495)

Lines 491 to 493

Belongs in the Discussion.

&gt; We have moved these lines to the discussion (Lines 513-518)

Figure 1 F and table 1

The numbers for Mas-AT differ from Utz et al. 2008. Why this difference?

&gt; The discrepancy in Mas-AT numbers is potentially due to differences in rearing conditions of the moths. Peptide expression is known to be sensitive to developmental stage, mating status, and hunger-state. However, all moths used in our study were naïve, unmated adults reared in separate male/female conditions between 2-6 days post-eclosion.

Discussion

Lines 497 to 499

Differentiating between the principal transmitter GABA and the neuropeptides would make it much easier to follow arguments throughout the Discussion centering on the peptides.

&gt; These instances have been fixed to clarify our arguments throughout the manuscript and discussion.

Lines 511 to 513

Why not have functionally defined LNs with overlapping peptide expression? For example,suppose that of 90 -RFamide LNs, 20 contain in addition AT, 20 AST-A and the rest AST-A plus say two more peptides. Are not these group functionally defined?

&gt; Yes, functionally defined LNs with overlapping peptide expression is likely the case. Our results suggest that functional classes cannot be defined by merely the expression of a single peptide due to this possibility. Our point is that the presence of single peptides should not be used to functionally define broad classes of neurons i.e. naming a class MIPergic or FMRFergic and assigning a function to this subset merely based on those peptide's presence is misleading. Ultimately, these peptides are heterogeneously expressed and likely many sub-classes of local interneurons within the larger population exist.

Line 514

What are “network consequences”?

&gt; We have changed this sentence to read “Furthermore, neuropeptide and GABAB receptors were expressed within each population of principal neuron type in the AL because ORNs, the influence of peptides are not segregated based on cell class-specific receptor expression.” (Lines 535-538)

From line 521

Citations that strenghten this argument:

e.g. Drosophila: Carlsson et al. 2010, Aedes aegypti: Siju et al. 2014, Tribolium: Binzer et al. 2014, Agrotis: Diesner et al. 2018)

&gt; The above citations were added (line 544)

Line 589 to 591

Meaning of this sentence is not clear.

&gt; This sentence has been changed to “Our results suggest the presence of single peptides should not be used to functionally define broad classes of neurons due to the stochastic heterogeneity of some transmitter co-expression patterns.”(Lines 619-622)

Line 618

“driven harder” is unclear.

&gt; This line has been to changed to “Finally, the network may need to be more strongly activated (i.e. by higher concentrations of odors or increased length of odor-stimuli) for LNs to release neuropeptides due to the different calcium binding affinities of distinct synaptotagmins associated with small clear vesicles and dense-core vesicles (Saraswati et al., 2007; Li et al., 2009).” (Lines 651-655)

Line 621 and 623

Is .....overall network “activity” and not “activation” meant here?

&gt;Line 611 has been changed to “overall network activity” (Line 633)

Lines 627 to 634

This well differentiated conclusion is not yet reflected, overall, in the paper.

&gt; We have expanded our discussion to further explain this conclusion

Lines 646 to 647

Correct but in only one aspect

&gt; We have changed this sentence to read “Ultimately, our results demonstrate that peptidergic modulation cannot be considered within the context of single neuropeptides as activation of any given LN results in a dynamic cocktail of modulators that have the potential to influence every level of olfactory processing within the AL.”
